# Trajectories of late‐life job insecurity and labor force participation: Associations with cognitive function and impairment in the Health and Retirement Study's harmonized cognitive assessment protocol

**DOI:** 10.1002/alz.71863

**Published:** 2026-09-23

**Authors:** Erika T. Beidelman, Xuexin Yu, Constance Beaufils, Rachel Donnelly, Mateo Farina, Amanda Sonnega, Lindsay C. Kobayashi, Yuan S. Zhang

**Affiliations:** ^1^ Robert N. Butler Columbia Aging Center Columbia University Mailman School of Public Health New York New York USA; ^2^ Department of Epidemiology Columbia University Mailman School of Public Health New York New York USA; ^3^ King's College London London UK; ^4^ Department of Sociology Vanderbilt University Nashville Tennessee USA; ^5^ Human Development & Family Sciences University of Texas at Austin Austin Texas USA; ^6^ Survey Research Center Institute for Social Research University of Michigan Ann Arbor Michigan USA; ^7^ Department of Epidemiology University of Michigan School of Public Health Ann Arbor Michigan USA; ^8^ Department of Sociomedical Sciences Columbia University Mailman School of Public Health New York New York USA

**Keywords:** cognitive function, employment, Health and Retirement Study, job insecurity, sequence analysis

## Abstract

**INTRODUCTION:**

Continued workforce participation may preserve later‐life cognition, yet many Americans face job insecurity and instability in later life, with potential consequences for cognitive aging.

**METHODS:**

Using Health and Retirement Study (HRS) Harmonized Cognitive Assessment Protocol data (*n* = 2418), we applied gender‐stratified sequence analyses to identify clusters of combined employment and job insecurity trajectories (1998 to 2014). We estimated associations with domain‐specific cognitive scores and impairment status in 2016 using weighted, multivariable‐adjusted regression.

**RESULTS:**

Six clusters were identified separately for women and men. Among women, self‐employed, early retirement, not in labor force, and secure‐to‐retirement trajectories were significantly associated with lower memory and executive function scores (*β* range = −0.13 to −0.32 standard deviation units) compared to secure employment. Among men, inconsistent employment was associated with poorer performance across most cognitive domains compared to secure employment. Employment instability and early retirement were associated with higher odds of cognitive impairment among women.

**DISCUSSION:**

These results underscore employment security as a potential protective factor for cognitive aging.

## Highlights

- Sequence analysis identified six distinct employment trajectories for men and women.
- Early retirement was linked to lower executive function and memory scores in women.
- Inconsistent employment was associated with lower cognitive scores in men.
- Stable employment clusters were associated with better cognitive health in late life.

## 1 BACKGROUND

Employment experiences during midlife have emerged as an important determinant of cognitive aging. Continued labor force participation has been consistently linked to positive cognitive outcomes in older age,^1^ whereas the absence of formal work experience and retirement have been associated with accelerated declines in cognition and memory function.^2^, ^3^, ^4^, ^5^ Evidence suggests that the cognitive benefits of continued employment are driven, at least in part, by sustained engagement in mentally stimulating work activities and the financial and psychosocial stability associated with employment.^6^, ^7^, ^8^, ^9^ Together, these findings suggest that sustained engagement in work throughout midlife may help preserve cognitive function, while employment instability may contribute to accelerated cognitive aging.

However, employment status alone does not fully capture the psychosocial dimensions of work, especially given the rise in insecure and unstable employment in recent decades.^10^ Older workers face heightened risks of employment instability and more limited reemployment opportunities compared to younger workers.^11^ In practice, many older adults remain employed under conditions of uncertainty and perceived vulnerability regarding job continuity.^12^ Perceived job insecurity, defined as an individual's subjective assessment of the likelihood of job loss, may fundamentally alter the cognitive and psychosocial experience of continued employment. Rather than fostering sustained cognitive investment, job insecurity may function as a chronic stressor that promotes psychological strain and disengagement from cognitively stimulating work tasks. Emerging empirical evidence supports this theory, demonstrating that perceived job insecurity is associated with reduced memory performance.^13^ This is further supported by evidence linking broader work‐related stressors (e.g., job demands) to adverse cognitive outcomes in older age.^14^, ^15^, ^16^, ^17^

A critical unanswered question is whether perceived job insecurity exerts an independent influence on cognitive aging beyond objective employment status. Prior studies typically assessed employment status and perceived job insecurity or work‐related stressors separately and did not examine how these stressful exposures unfolded alongside employment histories. As a result, it remains unclear how long‐term employment trajectories that capture both objective employment status and perceived job insecurity in midlife shape subsequent cognitive function and risk of cognitive impairment. Sequence analysis offers a critical methodological advantage in this regard, as it can represent the timing and ordering of employment states across the life course within a single trajectory framework,^17–19^ enabling a more comprehensive characterization of how employment experiences evolve over midlife when psychosocial factors are incorporated into employment states. In addition, the cognitive consequences of employment experiences likely depend on the timing and ordering of employment status across the life course. A more comprehensive characterization that incorporates perceived job insecurity as a chronic psychological stressor into employment histories will therefore advance our understanding of how work experiences from midlife to old age shape cognitive outcomes.

Importantly, exposure to long‐term employment instability and perceived job insecurity differs systematically by gender in exposure and likely its impact on cognitive health. Employment experiences across midlife are highly gendered, reflecting historical differences in labor force participation and caregiving responsibilities.^18^, ^19^ Women are more likely than men to experience prolonged periods of non‐participation in the formal labor force and interrupted employment trajectories, whereas men more often experience employment instability through involuntary job loss or inconsistent attachment to paid work.^18^ These differences are particularly relevant given that women bear a disproportionate burden of dementia in later life,^20^, ^21^ and recent evidence suggests that gendered patterns of labor force engagement may therefore have distinct implications for cognitive aging.^22^ These considerations underscore the necessity of examining gender‐specific employment trajectories and the gender‐specific associations with later‐life cognitive outcomes.

In this study, we examined the association of combined employment and perceived job insecurity trajectories in 1998 to 2014 with subsequent cognitive function and cognitive impairment among older men and women. By embedding subjective job insecurity within long‐term employment histories and examining gender‐specific patterns, this study advances existing literature and provides new insight into gendered employment characteristics from late midlife to later life that may be salient for cognitive aging.

**RESEARCH IN CONTEXT**

1. **Systematic review**: Prior research links continued labor force participation with positive cognitive outcomes in later life, while early retirement and unemployment are linked to accelerated decline. Although prior work has examined employment trajectories and cognition, none incorporated measures of perceived job insecurity, and few considered how employment histories differentially influenced cognitive health by gender.
2. **Interpretation**: Combined employment and job insecurity trajectories across midlife were strongly associated with later‐life cognitive performance. Gender‐stratified analyses revealed distinct patterns. Among women, early retirement and labor force non‐participation showed the strongest associations with cognitive deficits, whereas among men, inconsistent employment was the trajectory most consistently linked to poorer cognitive function.
3. **Future directions**: These findings suggest that employment instability in midlife could be a modifiable risk factor of dementia. Future research should examine how job characteristics contribute to these relationships and the effects of policies that support continued labor force participation among older adults.

## 2 METHODS

### 2.1 Study sample and setting

The Health and Retirement Study (HRS) is a longitudinal cohort study of health and aging in the US adult population aged 51 years and older and their spouses.^23^ Data collection for HRS began in 1992, with biennial interviews and regular refresher cohort replacements at the bottom end of its age range. HRS collects a wide variety of data through telephone and in‐person interviews, covering sociodemographic characteristics, employment information, physical health measures, cognitive function, and psychological well‐being.^23^ Data for this study came from the 1998 to 2014 core interview waves of the HRS and its Harmonized Cognitive Assessment Protocol (HCAP) sub‐study in 2016.

The HCAP delivers a comprehensive in‐person neuropsychological battery and an informant interview to a sub‐sample of HRS participants aged 65 years and older, extending well beyond the cognitive measures collected in the core surveys.^24^ We restricted the analytic sample to HCAP participants because our outcomes of interest were domain‐specific cognitive function and cognitive impairment. The HCAP collected more comprehensive and detailed neuropsychological data than the core interview, yielding domain‐specific scores for memory, executive function, language and fluency, and orientation, as well as an algorithmic classification of mild cognitive impairment (MCI) and dementia based on National Institute on Aging‐Alzheimer's Association (NIA‐AA) criteria.^25^

**FIGURE 1:**
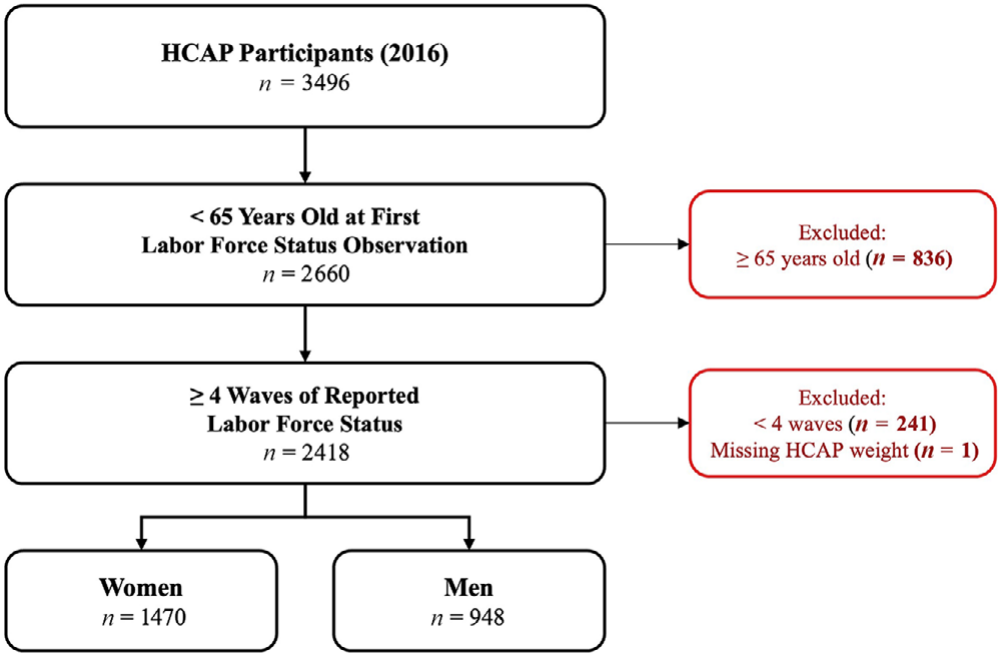
Sample selection flow chart for the analytic sample drawn from Health and Retirement Study (HRS) Harmonized Cognitive Assessment Protocol (HCAP) participants, 2016.

For models using cognitive impairment classification as the outcome, respondents were required to have either direct neuropsychological assessment or informant interviews. To ensure adequate longitudinal information for constructing employment trajectories, individuals were required to have at least four labor force status observations between 1998 and 2014 with corresponding perceived job insecurity data when formal employment was reported.^26^ Participants must have been enrolled in the HRS in 2008 or earlier to ensure they met this requirement. Additionally, we restricted our sample to participants who were less than 65 years old at their first observed labor force status measurement. These exclusions resulted in a final sample of *n* = 2418 (women: *n* = 1470; men: *n* = 948) HRS respondents (Figure 1). This study consisted of secondary analyses of previously collected, publicly available, de‐identified data and was determined to be exempt from Institutional Review Board review at Columbia University.

### 2.2 Exposure: employment state

For each wave in 1998 to 2014, participants were assigned to one of seven mutually exclusive employment states: (1) Employed‐Job Secure, (2) Employed‐Job Insecure, (3) Unemployed, (4) Retired, (5) Not in Labor Force, (6) Self‐Employed, and (7) Unreported. Employment states were derived from participants’ self‐reported labor force status and perceived job insecurity responses.

To classify participants, we first categorized respondents based on their formal employment status. Among individuals reporting formal employment, we used perceived job insecurity to distinguish between job‐secure and job‐insecure employment. Perceived job insecurity was measured for employed participants as the self‐reported likelihood of losing one's job in the next year (0 to 100 scale). Consistent with prior research, we dichotomized this measure at the 75th percentile.^27^ We used a pooled, gender‐specific 75th percentile where values equal to or above the threshold were coded as Employed‐Job Insecure while values below this threshold were coded as Employed‐Job Secure (see Appendix 1, Table A1.1 for comparison of wave‐specific and pooled thresholds).

For individuals not reporting employment, labor force status was used to assign employment state. Participants reporting that they were looking for work were coded as Unemployed, those reporting full retirement were coded as Retired, and those not working for reasons other than unemployment or retirement were coded as Not in Labor Force. Self‐employed respondents were not asked the job insecurity items. Because this structural nonresponse, distinct from missing labor force data, meant they could not be classified as Employed‐Job Secure or Employed‐Job Insecure, we retained self‐employment as its own state. For waves following enrollment where labor force status or job insecurity status (when employed) was missing, the employment state was coded as Unreported. The frequency of Unreported observations by wave and gender is reported in Appendix 1, Table A1.2. Part‐time and full‐time work were combined within the employed states. The distribution of part‐time work is described in Appendix 1, Tables A1.3a and A1.3b. These seven discrete states were then used to construct individual employment trajectories via sequence analysis (described below).

### 2.3 Outcome: cognitive domain scores and impairment status

We examined domain‐specific and global cognitive score data derived from the 2016 HRS HCAP. The domain‐specific scores included the memory, executive function, language and fluency, and orientation domains. Scores were analyzed on a standard deviation (z) scale. Memory, executive function, language and fluency, and global cognition scores were derived from the HCAP harmonized factor scores, standardized to a mean of 50 and standard deviation (SD) of 10 in the full HRS‐HCAP sample. These scores were then rescaled to z as (score − 50)/10. Orientation, available only as a 0 to 10 summary score, was z‐standardized using its HRS‐HCAP sample mean and SD. Standardization was performed on the pooled sample rather than within sex so that standardized association remained comparable in scale across the sex‐stratified models.

As an additional outcome, we examined the HCAP cognitive impairment classification. This was a binary indicator of probable MCI or dementia status based on the HCAP's classification algorithm. Participants who scored as having an impairment in at least two of the five cognitive domains as well as a self‐report of cognitive impairment from themselves or a proxy were classified as a probable dementia case by the algorithm.^25^ We included both MCI and dementia in our outcome due to small sample sizes for dementia classification alone.

### 2.4 Covariates

We used a set of covariates sourced from the HRS core interview to describe our sample, control for confounding, and handle differential mortality and attrition. These covariates included age at first wave, self‐rated health, depression score (Center for Epidemiologic Studies Depression Scale [CES‐D]), number of impairments in activities of daily living (ADLs), number of living children (0, 1 or 2, 3 or 4, or 5+), Stroke Belt residence (yes or no), marital status (married, never married, or divorced/separated/widowed), education level (college graduate, high school graduate, or less than high school/GED), cognitive z‐score at first wave, total household wealth (log‐transformed), and race/ethnicity (non‐Hispanic White, non‐Hispanic Black, or Hispanic/Other). The cognitive z‐score was derived from the HRS core 27‐point cognitive score.^28^ For this analysis, a participant's first wave was defined as their first HRS observation included in the employment trajectory analysis (i.e., starting year of their employment trajectory). We also defined covariates representing the total number of waves included in the participant's employment trajectory analysis as well as the wave at which each participant entered the HRS.

### 2.5 Sequence analysis

We performed gender‐stratified sequence analyses to define longitudinal employment trajectory clusters in 1998 to 2014. Participants contributed sequences of varying lengths dependent upon their year of entry into the HRS but were required to have at least four labor force status observations. Waves prior to a participant's study entry were treated as true missing values and left‐censored in the sequence analysis model. Missing states during a participant's enrollment were retained and assigned as the Unreported state.

Following a method described by Pacca et al. (2025), optimal matching (OM) was used to compute a person‐by‐person dissimilarity matrix for changes across the seven defined employment states over time.^26^ We fit the OM algorithm with a constant substitution cost matrix and an insertion/deletion cost of one. This choice allowed us to treat all transitions with equal cost and retain sensitivity to the timing and ordering of transitions across states. We evaluated the fit of two to 15 cluster solutions using the Calinski‐Harabasz index, Average Silhouette Width, and *R* ^2^ as well as inspection of meaningful group separation (Appendix 1, Table A1.4, and Figure A1.1). The six‐cluster solution was chosen for both men and women. Finally, Partitioning Around Medoids was applied to assign participants to their closest cluster match. The TraMineR and Cluster packages in R were used to perform sequence analysis.^29^, ^30^ Robustness of the six‐cluster solution to alternative cost and dissimilarity specifications is reported in Appendix 1, Table A1.5.

### 2.6 Inverse probability of censoring weighting

We constructed stabilized inverse probability of censoring weights (IPCWs) to account for differential loss to follow‐up over time due to study attrition and mortality between a participant's baseline HRS wave and the 2016 HCAP. For this analysis, baseline was defined as the participant's first eligible wave included in the sequence analysis. Among study‐eligible participants, 12.0% died and 22.5% were lost to follow‐up before the 2016 HCAP, while 65.5% were retained for the 2016 wave (Appendix 2, Table A2.1). The predictors for censoring were measured at baseline and included the baseline wave, gender, place of birth, education level, marital status, labor force status, self‐rated health, CES‐D score, household wealth quintile, and cognitive score. Missing predictor data were multiply imputed via chained equations (MICE) across 20 imputations. Within each imputed dataset we fit logistic regression models, separately, to predict mortality and attrition. Weights were averaged across the 20 imputations. Pooled odds ratios from the mortality and attrition weight models are reported in Appendix 2, Table A2.2. The mortality and attrition weights were multiplied to form a single stabilized censoring weight for each person. The combined IPCW for mortality and attrition was then multiplied by the HCAP 2016 survey weight and truncated at the first and 99th percentiles to produce the final analytic weights.

### 2.7 Regression analysis

We fit gender‐stratified linear regression models to assess the associations between employment trajectory cluster and each cognitive domain score. We also fit a gender‐stratified logistic regression model to assess the association between employment trajectory cluster and cognitive impairment status. All models were adjusted for a common set of baseline covariates measured at the respondent's first included trajectory wave: age at first wave, self‐rated health, depression score, ADLs, number of living children, region (Stroke Belt), education level, race/ethnicity, number of waves with non‐missing employment data, marital status, total household wealth, a binary indicator for HRS entry wave, and cognition at first wave. Adjustment for number of non‐missing employment waves accounted for differential observation lengths in employment histories. To address informative censoring due to mortality or study attrition between 1998 and 2016, all models applied IPCWs. Final estimates incorporated HCAP survey weights to ensure population‐representative inference consistent with HCAP sampling design. To aid interpretation of the domain associations, we benchmarked the cluster coefficients against the rate of cognitive aging by dividing the coefficient by the model's annual age coefficient for the same domain and sex. The benchmark represents the between‐person difference in years of cognitive aging based on cross‐sectional age differences at the 2016 assessment, not within‐person cognitive decline over time. All analyses were performed in R version 4.5.1.^31^

### 2.8 Sensitivity analyses

We performed several analyses to test the robustness of our results (detailed methods in Appendices^1^ and ^3^). To assess the robustness of the sequence analysis, we re‐derived the six‐cluster solution under alternative choices in exposure construction and clustering procedure. Because the job‐insecurity states depend on the dichotomization threshold, we re‐derived clusters using a wave‐specific 75th‐percentile threshold in place of the pooled within‐gender threshold used in the primary analysis. We also re‐estimated the OM distances under (1) a data‐derived (transition‐rate) substitution‐cost matrix in place of the constant‐cost matrix, (2) insertion/deletion costs of 0.5 and 2 in place of 1, and (3) alternative dissimilarity measures (spell‐based OM [OMspell] and the longest common subsequence [LCS]). Each alternative partition was compared with the primary six‐cluster solution using the adjusted Rand index (ARI) and the proportion of individuals who retained their primary trajectory cluster.

To assess the robustness of the associations, we refit the fully adjusted (Model 2) domain‐score and impairment models under three alternative cluster derivations. First, to address the potential for reverse causation arising from the short interval between the end of the exposure window (2014) and outcome ascertainment (2016), we truncated the observation window to end in 2012 and in 2010 then re‐derived the six‐cluster solution within each truncated window before refitting the models. Second, because self‐employed respondents were not administered the job insecurity items and could not be classified by insecurity status, we excluded respondents who were self‐employed in at least half of their observed waves and re‐derived the typology, which supported a five‐cluster solution. Third, because health‐related selection through cognitive or physical decline already present at baseline could drive both subsequent labor force exit and later cognitive outcomes, we refit the models on three restricted sub‐samples, holding cluster assignment fixed. Based on their first observed trajectory wave, the three sub‐samples excluded respondents with (1) probable dementia or disability, (2) probable cognitive impairment only, and (3) probable disability only. Baseline cognitive impairment was defined from the HRS 27‐point cognitive score (≤11 indicating any impairment and ≤6 indicating probable dementia)^28^ and disability as ≥1 self‐reported ADL limitation. Finally, for each statistically significant association, we computed E‐values for both the point estimate and the confidence limit nearer the null, quantifying the minimum association on the risk‐ratio scale that an unmeasured confounder would need with both the trajectory cluster and the cognitive outcome to fully explain away any observed associations.^32^

## 3 RESULTS

### 3.1 Sample characteristics

Across this sample of 1470 women and 948 men, the average ages at the first wave of observation were 56.7 (SD: 4.3) and 57.2 (SD: 3.8) years, respectively. Most participants identified as non‐Hispanic White and had at least a high school diploma (Table 1). Approximately 30% of women (*n* = 438) and 31% of men (*n* = 297) in our sample met the HCAP criteria for cognitive impairment (MCI or dementia) in 2016.

**TABLE 1:** Characteristics of analytic sample at first observed trajectory wave, stratified by gender: eligible 2016 Harmonized Cognitive Assessment Protocol (HCAP) participants (*N* = 2418).

Characteristic	Women *N* = 1470^a^	Men *N* = 948^a^
Age at first wave	56.7 (4.3)	57.2 (3.8)
Education level		
College graduate	313 (21%)	261 (28%)
High school graduate	823 (56%)	495 (52%)
Less than high school/GED	334 (23%)	191 (20%)
Race/ethnicity		
Hispanic or other	200 (14%)	113 (12%)
Non‐Hispanic Black	244 (17%)	101 (11%)
Non‐Hispanic White	1026 (70%)	734 (77%)
Marital status		
Married	1075 (73%)	808 (85%)
Never married	44 (3.0%)	29 (3.1%)
Separated/divorced/widowed	350 (24%)	110 (12%)
Total household wealth (USD)	317,665 (696,760)	342,134 (652,282)
Cognitive score at first wave (z‐score)	0.22 (0.94)	0.00 (0.91)
Depressive symptoms (CES‐D, 0 to 8)	1.45 (2.02)	1.07 (1.68)
Self‐rated health		
Excellent	363 (25%)	260 (27%)
Very good	443 (30%)	291 (31%)
Good	387 (26%)	272 (29%)
Fair	194 (13%)	94 (9.9%)
Poor	83 (5.6%)	31 (3.3%)
Any ADL limitation	141 (9.6%)	59 (6.2%)
Number of living children		
0	86 (5.9%)	62 (6.6%)
1 or 2	536 (37%)	349 (37%)
3 or 4	538 (37%)	368 (39%)
5+	303 (21%)	160 (17%)
Residence in Stroke Belt	646 (44%)	363 (38%)
Employment trajectory cluster		
Predominantly job secure	257 (17%)	133 (14%)
Secure employment to retirement	341 (23%)	198 (21%)
Predominantly job insecure	148 (10%)	119 (13%)
Predominantly self‐employed	112 (7.6%)	122 (13%)
Early retirement	413 (28%)	195 (21%)
Inconsistent employment	—	181 (19%)
Not in labor force	199 (14%)	—
Any cognitive impairment (in 2016)	438 (30%)	297 (31%)

^a^Mean (SD); *n* (%)

### 3.2 Employment trajectory clusters

**FIGURE 2 alz71863-fig-0002:**
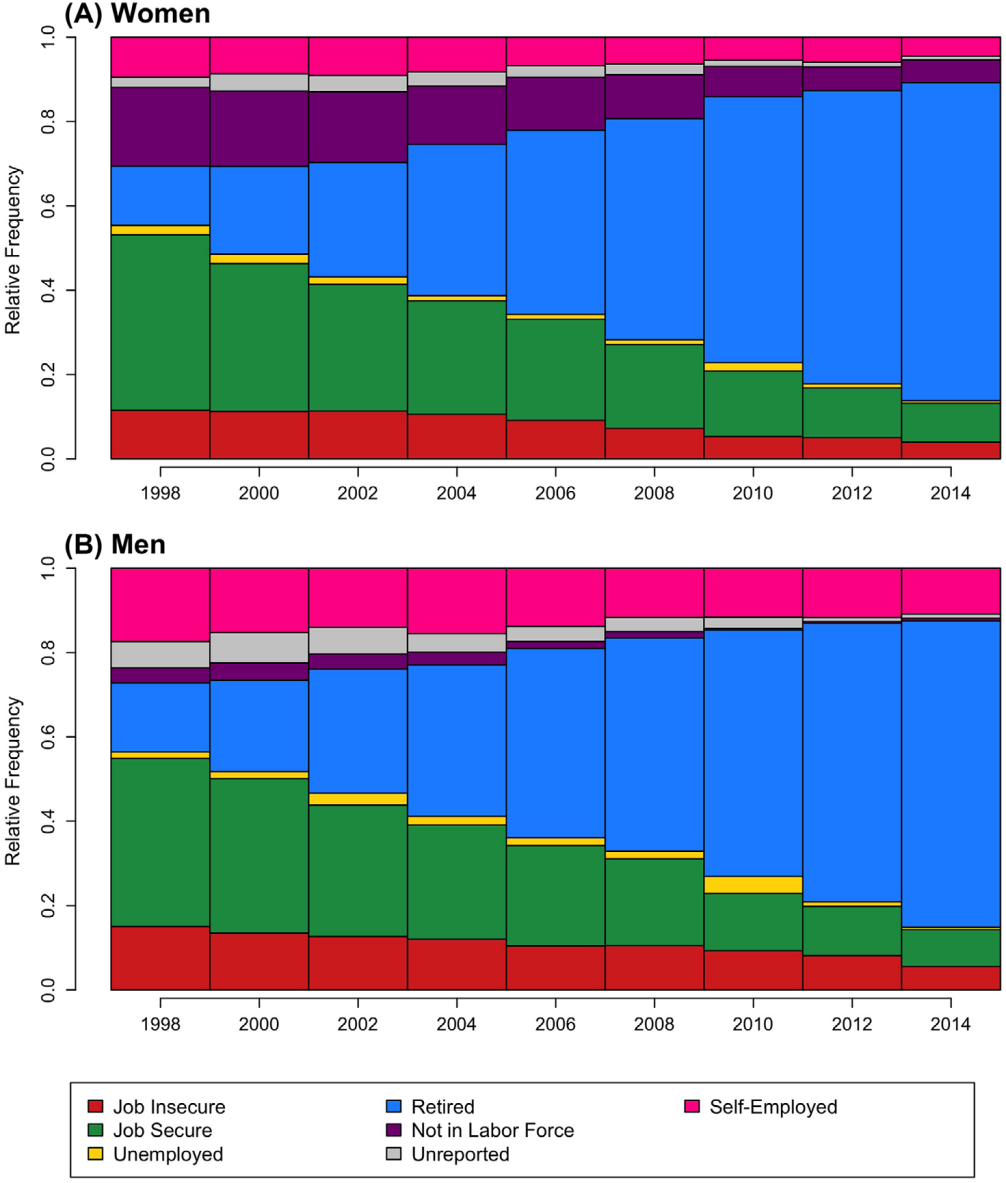
Cross‐sectional distribution of job insecurity states by survey wave (1998 to 2014) among women (A) and men (B), HRS sequence analysis sample (*n* = 2418). Employment states were derived from self‐reported labor force status. Among employed person‐waves, perceived job insecurity was dichotomized at the pooled within‐gender 75th percentile of the self‐reported probability of job loss (Job Insecure ≥ threshold; Job Secure < threshold). “Unreported” denotes missing labor‐force information during observation. Bars show distribution of states among respondents observed at each wave.

**FIGURE 3 alz71863-fig-0003:**
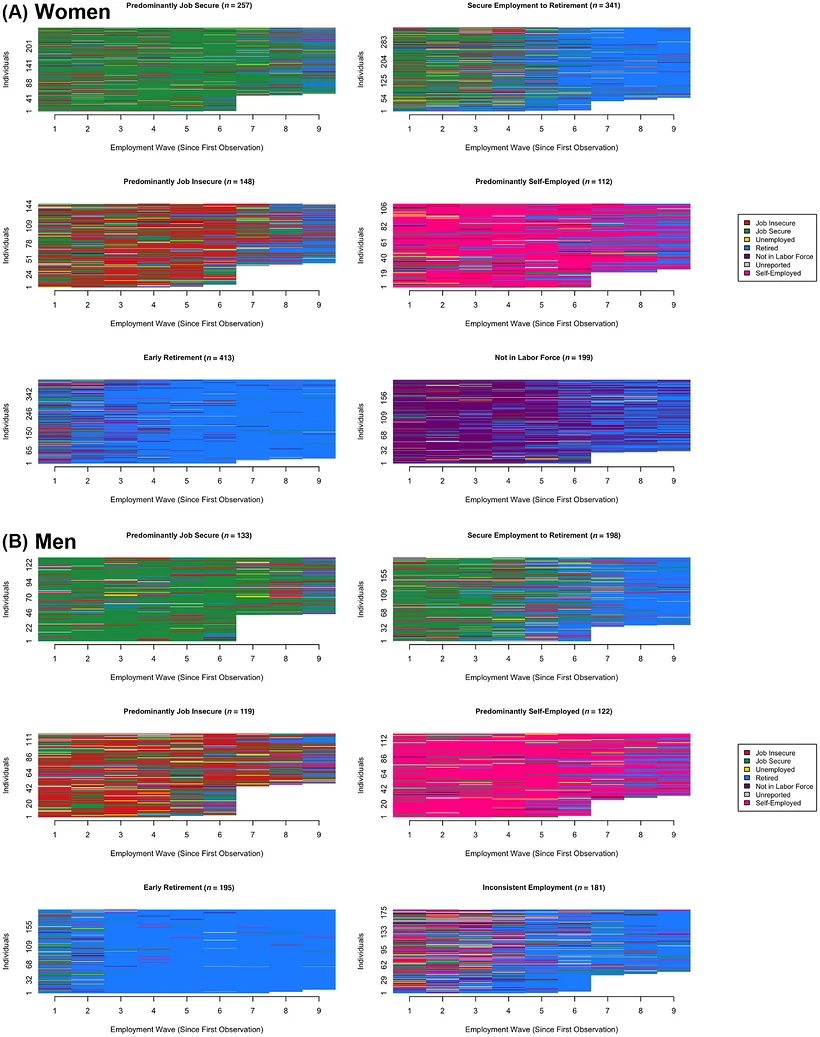
Individual job insecurity trajectories (1998 to 2014) within each cluster, women (A) and men (B), HRS sequence analysis sample (*n* = 2418).

The patterns of employment states defining the sequence analysis period are illustrated in Figure 2, and gender‐specific clusters are depicted in Figure 3. Our observation waves spanned from high labor force participation in the early waves to predominantly retired ages in the later waves, enabling us to capture employment trajectories over the ages at which individuals exit the labor market. Sequence analysis identified six distinct employment trajectory clusters for both women and men (see Appendix 1 for the cluster fit statistics). The Predominantly Job Secure cluster (women: *n* = 257; men: *n* = 133), used as the reference group, reflected sustained, primarily secure employment. The Secure to Retirement transition cluster (women: *n* = 341; men: *n* = 198) was characterized by early job‐secure employment followed by movement into retirement. The Predominantly Job Insecure cluster (women: *n* = 148; men: *n* = 119) reflected consistent employment marked by persistent job insecurity. The Predominantly Self‐Employed cluster (women: *n* = 112; men: *n* = 122) consisted of individuals with consistent self‐employment, while the Early Retirement cluster (women: *n* = 413; men: *n* = 195) consisted of individuals who retired at the beginning of the employment trajectory period. Additionally, one gender‐specific cluster emerged for each gender. Among women, a Not in the Labor Force cluster (*n* = 199) reflected long periods of non‐participation[Fig alz71863-fig-0002], [Fig alz71863-fig-0003] in the labor force for reasons other than retirement or unemployment. Among men, an Inconsistent Employment cluster (*n* = 181) reflected frequent shifts between working, unemployed, and retired states.

### 3.3 Domain‐specific cognitive scores

Among women, all employment trajectory clusters, except the Predominantly Job Insecure cluster, were associated with cognitive deficits concentrated in executive function, memory, and global cognition compared to the Predominantly Job Secure cluster (Table 2). Within these domains, the largest differences were observed for the Early Retirement and Not in Labor Force clusters. Women who retired early had memory scores that were on average 0.30 SD (95% CI: −0.46, −0.13) lower than women who were predominantly job secure, while memory scores for women who were not in the labor force were on average 0.32 SD units (95% CI: −0.50, −0.14) lower. Self‐employed women were also broadly affected, with significantly lower executive function, memory, language and fluency, and global cognition scores. They were also the only women's cluster with a significant language and fluency deficit (−0.29 SD units, 95% CI: −0.50, −0.08). Secure‐to‐Retirement women showed more selective deficits, limited to memory and executive function. Language and fluency was otherwise largely preserved across clusters, and orientation deficits were confined to the Not in Labor Force and Early Retirement clusters.

**TABLE 2 alz71863-tbl-0002:** Adjusted differences in 2016 HCAP cognitive domain z‐scores by job insecurity trajectory cluster (1998 to 2014), among women (reference: Predominantly Job Secure).

	Executive function	Memory	Language and fluency	Orientation	Global cognition
	*B (95% CI)*	*B (95% CI)*	*B (95% CI)*	*B (95% CI)*	*B (95% CI)*
Predominantly job secure	*Reference*	*Reference*	*Reference*	*Reference*	*Reference*
Secure to retirement	−0.13 (−0.25, −0.01) ^a^	−0.19 (−0.33, −0.05) ^a^	−0.04 (−0.21, 0.13)	−0.00 (−0.10, 0.09)	−0.10 (−0.21, 0.01)
Predominantly job insecure	−0.01 (−0.16, 0.14)	−0.02 (−0.19, 0.15)	0.12 (−0.09, 0.33)	0.08 (−0.05, 0.20)	0.08 (−0.07, 0.22)
Predominantly self‐employed	−0.15 (−0.30, −0.01) ^a^	−0.24 (−0.42, −0.07) ^a^	−0.29 (−0.50, −0.08) ^a^	−0.06 (−0.21, 0.09)	−0.19 (−0.36, −0.02) ^a^
Early retirement	−0.21 (−0.35, −0.07) ^a^	−0.30 (−0.46, −0.13) ^a^	−0.14 (−0.33, 0.04)	−0.17 (−0.33, −0.00) ^a^	−0.18 (−0.32, −0.03) ^a^
Not in labor force	−0.25 (−0.39, −0.11) ^a^	−0.32 (−0.50, −0.14) ^a^	−0.13 (−0.34, 0.07)	−0.19 (−0.34, −0.05) ^a^	−0.23 (−0.39, −0.07) ^a^

*Note*: Estimates are weighted by HCAP 2016 survey weights and inverse probability of censoring weights, adjusted for age, education, race/ethnicity, marital status, household wealth, HRS entry wave, number of observed employment waves, cognition, self‐rated health, depressive symptoms, activities of daily living, number of living children, and stroke‐belt residence. All covariates were measured at baseline (first included trajectory wave).

^a^ *p* < 0.05.

**FIGURE 4 alz71863-fig-0004:**
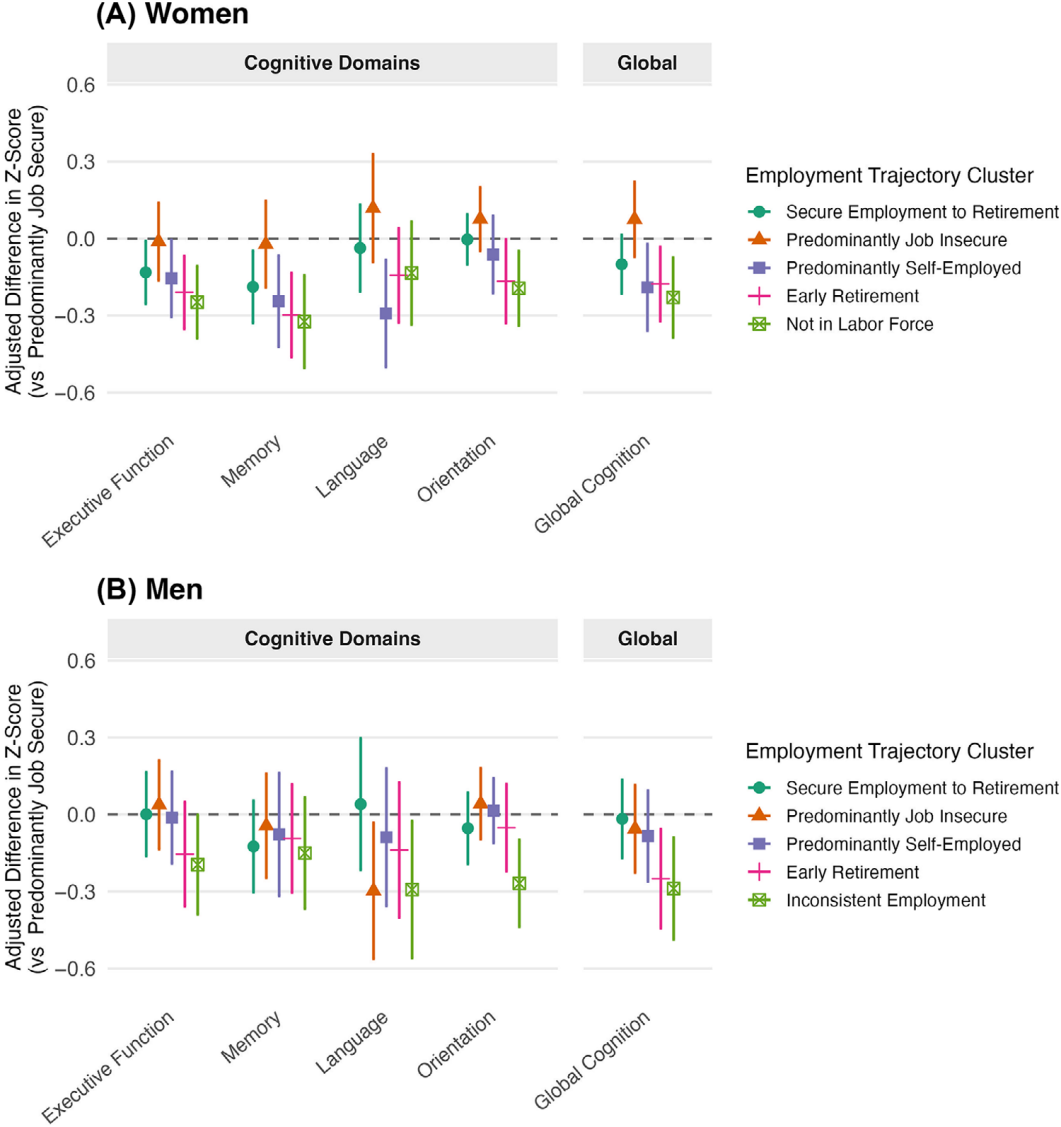
Adjusted differences in cognitive domain z‐scores (95% CI) for each employment‐trajectory cluster relative to the predominantly job secure reference, from the fully adjusted model, among women (A) and men (B). Points show adjusted difference in domain z‐scores (95% CI) for each cluster relative to Predominantly Job Secure reference represented by the dashed line at 0. Intervals excluding 0 indicate *p* < 0.05. Estimates are from survey‐weighted linear regression weighted by HCAP 2016 survey weights and inverse probability of censoring weights, adjusted for age, education, race/ethnicity, marital status, household wealth, HRS entry wave, number of observed employment waves, baseline cognition, self‐rated health, depressive symptoms, activities of daily living, number of living children, and stroke‐belt residence. Global cognition (a composite) is shown separately from domain‐specific outcomes.

In contrast to women, only the Inconsistent Employment trajectory cluster was consistently associated with lower cognitive domain scores among men (Table 3). Inconsistently employed men scored significantly lower across the executive function (*β* = −0.20, 95% CI: −0.39, −0.00), language and fluency (*β* = −0.29, 95% CI: −0.56, −0.03), and orientation (*β* = −0.27, 95% CI: −0.44, −0.10) domains compared to men in the Predominantly Job Secure cluster. Additionally, both the Early Retirement and Inconsistent Employment clusters were associated with global cognition scores that were 0.25 (95% CI: −0.44, −0.06) and 0.29 SD units (95% CI: −0.49, −0.09) lower than the Predominantly Job Secure cluster, respectively. Unlike the pattern among women, memory was not significantly lower for any male cluster. The Predominantly Job Insecure cluster showed an isolated significant language and fluency deficit (*β* = −0.30, 95% CI: −0.56, −0.03). Figure 4 plots the cognitive score differences and 95% confidence intervals by employment trajectory cluster for women (Panel A) and men (Panel B), illustrating the broader pattern of differences across clusters and domains.

**TABLE 3 alz71863-tbl-0003:** Adjusted differences in 2016 HCAP cognitive domain z‐scores by job insecurity trajectory cluster (1998 to 2014), among men (reference: Predominantly Job Secure).

	Executive function	Memory	Language & Fluency	Orientation	Global cognition
	*B (95% CI)*	*B (95% CI]*	*B (95% CI)*	*B (95% CI)*	*B (95% CI)*
Predominantly job secure	*Reference*	*Reference*	*Reference*	*Reference*	*Reference*
Secure to retirement	0.00 (−0.16, 0.16)	−0.12 (−0.30, 0.05)	0.04 (−0.22, 0.30)	−0.05 (−0.19, 0.09)	−0.02 (−0.17, 0.14)
Predominantly job insecure	0.04 (−0.13, 0.21)	−0.04 (−0.25, 0.16)	−0.30 (−0.56, −0.03) ^a^	0.04 (−0.10, 0.18)	−0.06 (−0.23, 0.11)
Predominantly self−employed	−0.01 (−0.19, 0.17)	−0.08 (−0.32, 0.16)	−0.09 (−0.36, 0.18)	0.01 (−0.11, 0.14)	−0.08 (−0.26, 0.09)
Early retirement	−0.15 (−0.36, 0.05)	−0.09 (−0.30, 0.12)	−0.14 (−0.40, 0.12)	−0.05 (−0.22, 0.12)	−0.25 (−0.44, −0.06) ^a^
Inconsistent employment	−0.20 (−0.39, −0.00) ^a^	−0.15 (−0.37, 0.07)	−0.29 (−0.56, −0.03) ^a^	−0.27 (−0.44, −0.10) ^a^	−0.29 (−0.49, −0.09) ^a^

*Note*: Estimates are weighted by HCAP 2016 survey weights and inverse probability of censoring weights, adjusted for age, education, race/ethnicity, marital status, household wealth, HRS entry wave, number of observed employment waves, cognition, self‐rated health, depressive symptoms, activities of daily living, number of living children, and stroke‐belt residence. All covariates were measured at baseline (first included trajectory wave).

^a^ *p* < *0.05*.

### 3.4 Cognitive impairment status

Among women, the Early Retirement and Not in the Labor Force clusters were associated with nearly twice the odds of any cognitive impairment compared to the[Table alz71863-tbl-0002], [Table alz71863-tbl-0003] Predominantly Job Secure cluster (adjusted odds ratio [aOR] = 1.97, 95% CI: 1.19, 3.27 and aOR = 1.80, 95% CI: 1.03, 3.16, respectively)[Fig alz71863-fig-0004] (Table 4). In contrast, the Predominantly Job Insecure cluster compared to the Predominantly Job Secure cluster was associated with reduced odds of cognitive impairment (aOR = 0.46, 95% CI: 0.23, 0.92). Among men, the Inconsistent Employment cluster was associated with twice the odds cognitive impairment (aOR = 2.02, 95% CI: 1.06, 3.83) compared to the Predominantly Job Secure cluster.

**TABLE 4 alz71863-tbl-0004:** Adjusted odds ratios for 2016 HCAP cognitive impairment classification (mild cognitive impairment or dementia) by employment and perceived job insecurity trajectory cluster (1998 to 2014), stratified by gender (reference: Predominantly Job Secure).

	Women	Men
	*aOR (95% CI)*	*aOR (95% CI)*
Predominantly Job Secure	*Reference*	*Reference*
Secure to Retirement	1.36 (0.83, 2.24)	1.45 (0.78, 2.69)
Predominantly Job Insecure	0.46 (0.23, 0.92) ^a^	1.37 (0.68, 2.77)
Predominantly Self‐Employed	1.87 (0.98, 3.59)	0.89 (0.43, 1.81)
Early Retirement	1.97 (1.19, 3.27) ^a^	1.48 (0.74, 2.96)
Not in Labor Force	1.80 (1.03, 3.16) ^a^	–
Inconsistent Employment	–	2.02 (1.06, 3.83) ^a^

*Note*: Estimates are weighted by HCAP 2016 survey weights and inverse probability of censoring weights, adjusted for age, education, race/ethnicity, marital status, household wealth, HRS entry wave, number of observed employment waves, cognition, self‐rated health, depressive symptoms, activities of daily living, number of living children, and stroke‐belt residence. All covariates were measured at baseline (first included trajectory wave). Cognitive impairment includes both mild cognitive impairment and dementia classifications.

*p < *0.05*.

### 3.5 Sensitivity analyses

Our findings were robust across the sensitivity analyses (Appendices 1 and 3). The six‐cluster typology was reproduced across the alternative cost and dissimilarity specifications, with 77% to 100% of individuals retaining their primary trajectory archetype. The original observed cognitive associations remained stable. The sole exception was the women's Predominantly Job Insecure cluster, which did not persist as a distinct group under data‐derived (transition‐rate) substitution costs. All other statistically significant associations were preserved. Re‐derivation of the solution on the truncated exposure windows recovered the primary archetypes for 90% (1998 to 2012) and 82% (1998 to 2010) of individuals, and the exclusion of predominantly self‐employed respondents recovered 99%. In each case, the associations between the refit clusters and the cognitive outcomes were consistent in direction and magnitude, with the majority of the primary associations retaining significance (Appendix 3, Tables A3.1 and A3.2). Additionally, several associations were as strong or stronger despite the longer exposure–outcome interval.

Excluding respondents with prevalent impairment or disability at baseline (∼8% with any probable cognitive impairment and <1% with probable dementia) left the cognitive domain estimates essentially unchanged (Appendix 3, Tables A3.3, A3.4, and A3.5). The cognitive impairment associations were directionally stable but attenuated in the smaller restricted samples. In particular, the women's Early Retirement and Not in Labor Force impairment associations weakened when respondents with baseline disability were excluded, whereas the corresponding memory associations persisted. E‐values for the primary associations were approximately 2.0 to 2.3 for point estimates (Appendix 3, Table A3.6).

## 4 DISCUSSION

We leveraged longitudinal data from an ongoing, nationally representative study of older Americans to understand how employment trajectories beginning in late midlife were associated with cognitive outcomes in later life. By jointly characterizing labor force participation and perceived job insecurity over 16 years, this study captures the co‐evolution of objective employment states and subjective work experiences in a way that prior studies have not. We found that long‐term employment trajectories across midlife were meaningfully associated with both cognitive scores and odds of cognitive impairment in later life. We also observed clear gender differences in how employment trajectories related to cognitive performance. Still, across both men and women, trajectories characterized by early labor force exits and non‐participation in the formal labor market were consistently associated with cognitive deficits compared to trajectories defined by stable employment. These differences were substantial. Relative to those with stable and secure employment, the global cognitive deficit for inconsistently employed men corresponded to approximately 7 years of cognitive aging while the memory deficits for women who retired early or were not in the labor force equaled approximately 6 years (Appendix 3, Table A3.7).

Interestingly,[Table alz71863-tbl-0004] our results suggest that employment continuity, rather than subjective job security, may be the more protective factor for cognitive aging. Contrary to our hypothesis, participants who maintained stable employment despite reporting persistent job insecurity did not exhibit cognitive deficits compared to stable, job‐secure participants. This contrasts with prior longitudinal work showing that perceived job insecurity was associated with poorer baseline memory performance.^13^ One possibility is that individuals reporting persistent insecurity during the study period may have experienced job insecurity earlier in life as well, and this prior exposure may have already affected baseline cognitive function. This may also suggest that some of the disadvantages attributed to job insecurity in earlier studies may reflect underlying employment or economic instability in addition to the insecurity itself. Critically, most of our job‐insecure cluster remained employed long term, separating the perception of insecurity from insecurity that ended in job loss. If anything, this cluster fared marginally better. This cluster's domain estimates, though non‐significant, were uniquely at or above the job‐secure reference and it showed significantly lower odds of cognitive impairment. Plausible explanations for this relationship exist. For instance, insecurity could prompt greater effort and engagement at work to reduce the risk of job loss, whereas security could invite disengagement. However, given the cluster's small size and instability under alternative specifications, we treat these results cautiously as a direction for further study. In contrast, cognitive deficits and greater impairment risk were concentrated instead among those who exited the labor force early, stayed outside it, or shifted frequently across employment states. This implicates objective employment disruption, rather than perceived insecurity, as the greater cognitive risk in midlife to later life.

Our findings build on and extend prior work demonstrating that unstable employment, marked by unemployment and illness, is associated with higher risk of cognitive impairment and dementia.^33^, ^34^ Our results also align with recent evidence suggesting that women with a lack of formal work experience across the life course exhibit steeper cognitive decline compared to women with formal work experience.^3^ While prior work focused exclusively on transitions between objective employment states, our sequence analysis approach uniquely incorporated the subjective dimension of perceived job insecurity. By integrating this psychosocial dimension, we showed that stable employment of any kind was associated with the most favorable cognitive profiles, underscoring the importance of labor market continuity for cognitive aging.

Several mechanisms may explain our observed associations. Sustained engagement in paid work may support the accumulation and maintenance of cognitive reserve by providing regular cognitive stimulation, problem‐solving demands, and socially complex interactions that are protective against age‐related cognitive decline.^7^, ^35^ Consistent with this framework, occupations involving greater mental demands or higher complexity have been associated with better cognitive functioning and a lower risk of dementia.^6^, ^36^, ^37^, ^38^, ^39^ When employment is interrupted or exited prematurely, these sources of stimulation are reduced or lost altogether, potentially accelerating cognitive decline.^4^, ^5^ Employment disruptions are also typically accompanied by negative economic consequences, which are also associated with reduced cognitive scores and increased dementia risk.^40^, ^41^ In contrast, perceived job insecurity does not necessarily entail disengagement from cognitively demanding tasks and economic disruptions if individuals remain actively employed. However, it may still operate as a chronic psychological stressor, with plausible downstream effects on cognition through stress‐related neuroendocrine and inflammatory dysregulation.^42^, ^43^, ^44^

The associations we observed were more consistent and precise among women than men. This partly reflects statistical power, as the male sub‐sample was smaller and produced wider confidence intervals, but it is also consistent with the gendered structure of midlife employment.^22^ Women more often experience prolonged periods outside the formal labor force and interrupted employment trajectories, frequently tied to caregiving and childrearing, and it was precisely these early‐exit and non‐participation trajectories that were most strongly associated with poorer later‐life cognition. Adjusting for the number of living children did not alter these associations, suggesting they are not simply a function of family size. Nonetheless, this pattern aligns with prior HRS evidence that sustained paid work across women's early and mid‐adult lives predicts slower later‐life memory decline.^45^ More granular caregiving measures, such as time spent in unpaid care, are collected only intermittently and largely after employment trajectories begin. Direct caregiving intensity therefore could not be modeled, and residual confounding by these gendered life‐course factors cannot be excluded.

The two cognitive outcomes were broadly concordant. The clusters showing the largest deficits on the continuous domain scores were also those with higher odds of algorithmically classified cognitive impairment. Because the continuous scores and the categorical classification are derived and scaled differently, their agreement on the same high‐risk clusters strengthens confidence that these trajectories mark genuinely poorer cognitive health rather than an artifact of one outcome definition. The two also remain complementary. Domain scores like memory capture graded differences across the full distribution and are sensitive to the subtle decline that emerges earlier in the cognitive aging process, whereas the impairment classification reflects crossing a later, clinically defined threshold.^46^ This likely explains why several clusters with modest domain‐score deficits were not significantly associated with impairment.

This study has several strengths, including the use of a national study with over 16 years of detailed employment data, the integration of perceived job insecurity into long‐term employment trajectories, and the use of a comprehensive neuropsychological protocol to assess cognitive function and probable cognitive impairment status. Nevertheless, several limitations warrant discussion. Employment states and job insecurity were self‐reported, which may introduce misclassification and measurement error. We also could not model work intensity within our sequences. Part‐time work was rare among men, so adding it as a separate state would leave several trajectory groups too sparse to estimate. This may matter most for women's trajectories, where part‐time work often reflects caregiving. Relatedly, we lacked data to account for other work‐related stressors, such as job strain and workplace discrimination. Because these exposures are plausibly correlated with both adverse employment trajectories and cognitive aging, their omission introduces the potential for unmeasured confounding. Although we adjusted for baseline cognitive function and health and used censoring weights to account for differential attrition, residual bias due to health selection remains possible. Employment exits and instability may partly reflect early sub‐clinical cognitive or health decline. Restricting outcomes to the HCAP sub‐sample, though necessary to obtain domain‐specific cognitive measures and a validated impairment classification, reduced our sample size and power relative to the full HRS core. Consequently, some clusters contained relatively few individuals meeting impairment criteria, yielding wide confidence intervals and limited precision for those estimates. Finally, cognitive impairment classification in HCAP is algorithmic and inherently imperfect, which may lead to some degree of outcome misclassification. Yet, prior studies showed that cognitive impairment prevalence estimates from the HRS‐HCAP were comparable to those derived from other national studies of health and aging,^47^ suggesting that probable cognitive impairment status is likely reliable for our analysis.

Despite these limitations, our findings carry meaningful implications for dementia prevention and public health policy. Employment continuity across midlife may represent a modifiable factor associated with cognition decades later. Policies supporting sustained labor force attachment, reducing involuntary job transitions, and expanding access to cognitively engaging employment throughout midlife may help promote healthier cognitive aging. Individuals with histories of employment instability or extended labor force absence may represent key groups for early identification and preventive cognitive health interventions. Future studies should further investigate how other job characteristics such as job demands and occupational environment interact with long‐term employment trajectories to shape cognitive reserve and dementia risk. Research capturing job insecurity across a broader span of the life course would also help clarify whether and how earlier experiences of insecurity contribute to the employment disruptions and cognitive outcomes observed in our study. In conclusion, long‐term employment trajectories constitute an important life‐course exposure linked to late‐life cognitive health, and addressing gendered and structural differences in employment patterns may offer new opportunities for promoting cognitive resilience and reducing dementia disparities.

## CONFLICT OF INTEREST STATEMENT

The authors declare no conflicts of interest. Author disclosures are available in the Supporting Information.

## CONSENT STATEMENT

The Health and Retirement Study obtained oral informed consent from all participants prior to interviews.

## Supporting information




Supporting Information

Supporting Information
