# Supplementary material for: Trajectories of late‐life job insecurity and labor force participation: Associations with cognitive function and impairment in the Health and Retirement Study's harmonized cognitive assessment protocol
Source: Alzheimers Dement. 2026 Sep 23;22(9):e71863. doi: 10.1002/alz.71863 (PMC13598547; doi:10.1002/alz.71863)
Supplement: Supplementary file 2 [file ALZ-22-e71863-s001.docx]

**Appendix 1. Sequence Analysis Sensitivity and Robustness**

As described in the Methods, employment–insecurity trajectories (1998–2014) were derived by optimal matching (OM) and PAM clustering. In this supplemental appendix, we detail the choice of cluster number and its robustness. Perceived job insecurity (self-reported 0–100 probability of job loss) was dichotomized at the pooled within-gender 75th percentile (≥25 for women, ≥20 for men). We pooled across waves so that an identical reported probability received the same classification regardless of survey year. A wave-specific 75th percentile, by contrast, varied substantially over time (range: 10–40) and could classify identical probabilities inconsistently. This choice had little bearing on the resulting clusters. The pooled and wave-specific cutoffs agreed on 95.7% of employed person-waves, and re-deriving clusters under the wave-specific cutoff closely reproduced the primary six-cluster solution. Wave-specific thresholds are reported in Table A1.1.

Approximately 2.6% of women’s employment observations and 4.1% of men’s employment observations were classified as Unreported with rates declining over time (Table A1.2). Coding intermittently missing labor-force observations as a distinct “Unreported” state, rather than dropping affected respondents or imputing status, follows established practice in life-course sequence analysis. Among employed (job secure or job insecure) person-waves, part-time work was substantially more common for women than men (17.3% vs. 4.9%; 12.3% overall), and 35.9% of ever-employed women worked part-time in at least one wave versus 14.4% of men (Table A1.3). Because part-time and full-time work were combined within the employed states, the typology does not distinguish work intensity. Given the small share of part-time waves among men and the modest analytic sample, we did not model part-time status as a separate state.

Our primary criterion for selecting the number of clusters was to retain every trajectory type that was both substantively meaningful and represented enough participants for stable estimation. By this standard, six clusters were optimal in both sexes. Through *k* = 6, each successive cluster corresponded to a distinct, interpretable employment–insecurity trajectory of an adequate size (smallest men’s cluster: n = 119 and women’s cluster: n = 112). Beyond six clusters, additional groups no longer satisfied both conditions simultaneously. The seventh cluster did not isolate a new, substantively distinct trajectory but instead redistributed members drawn from several existing groups, and further subdivision produced increasingly small clusters. By *k* = 8, a group of only 35 individuals emerged among men. In other words, any additional division capable of revealing a genuinely new pattern would necessarily have yielded clusters too small to support adequately powered estimation. We therefore retained a common six-cluster solution for both sexes, which also allowed the two typologies and their shared archetypes to be compared.

This substantive choice was corroborated by a panel of internal cluster-quality indices computed for *k* = 2 to *k* = 15 by sex. The cluster-quality indices included point-biserial correlation (PBC), Hubert's gamma (HG), average silhouette width (ASW), Calinski–Harabasz index (CH), explained variance (R²), and Hubert's C (HC) (Table A1.4, Figure A1.1). Among women, these indices directly supported six clusters. Both PBC (0.60) and ASW (0.34) reached their maxima at *k* = 6, HG was high (0.87), and HC was near its minimum. Among men, indices that reward compactness favored a more parsimonious three-cluster solution. Nonetheless, cluster quality at *k* = 6 remained reasonable rather than degraded. PBC declined only modestly from its peak (0.56 vs. 0.63 at *k* = 3), HG stayed high (0.84), and HC remained low (0.10, near its *k* = 5 minimum of 0.09). Because a three-cluster solution for men would collapse meaningful trajectory types, the interpretability and cross-sex comparability afforded by six clusters outweighed the marginally greater statistical compactness of a smaller solution.

The chosen six-cluster solution and its associations with cognition proved robust across alternative dissimilarity specifications and cluster solutions. The six-cluster typology was robust to the substitution cost matrix (constant vs. transition-rate), the indel cost (0.5–2), and the dissimilarity measure (OM, OMspell, OMslen, LCS), with 77–100% of individuals retaining their trajectory archetype across specifications (Table A1.5). The one exception, worth stating explicitly, was the women’s Predominantly Job Insecure cluster, which did not persist as a distinct group under data-driven (transition-rate) substitution costs. Its members redistributed into the Job Secure and Early Retirement clusters. This is consistent with perceived insecurity acting as a subtle overlay on otherwise stable employment rather than a sharply separated trajectory. Notably, this was the null cluster for women. The significant women’s associations were unaffected.

Table A1.1. Perceived job-insecurity 75th-percentile threshold (0-100 scale) among all enrolled employed person-waves (1998-2014), by survey wave and gender

| **Wave** | **Women** | **Men** |
| --- | --- | --- |
| 1998 | 10.0 | 10.00 |
| 2000 | 20.0 | 20.00 |
| 2002 | 25.0 | 20.00 |
| 2004 | 30.0 | 20.00 |
| 2006 | 25.0 | 20.00 |
| 2008 | 27.5 | 25.00 |
| 2010 | 30.0 | 38.75 |
| 2012 | 40.0 | 25.00 |
| 2014 | 25.0 | 20.00 |
| Pooled (primary) | 25.0 | 20.00 |

Table A1.2. Number and percentage of person-waves (1998-2014) coded as 'Unreported', by survey wave and gender

| **Gender** | **Wave** | **Person-waves** | **Unreported** | **% Unreported** |
| --- | --- | --- | --- | --- |
| Female | 1998 | 1,241 | 28 | 2.3 |
| Female | 2000 | 1,265 | 54 | 4.3 |
| Female | 2002 | 1,281 | 49 | 3.8 |
| Female | 2004 | 1,460 | 46 | 3.2 |
| Female | 2006 | 1,466 | 47 | 3.2 |
| Female | 2008 | 1,470 | 44 | 3.0 |
| Female | 2010 | 1,470 | 22 | 1.5 |
| Female | 2012 | 1,470 | 18 | 1.2 |
| Female | 2014 | 1,470 | 15 | 1.0 |
| Male | 1998 | 735 | 48 | 6.5 |
| Male | 2000 | 751 | 54 | 7.2 |
| Male | 2002 | 765 | 56 | 7.3 |
| Male | 2004 | 940 | 46 | 4.9 |
| Male | 2006 | 946 | 43 | 4.5 |
| Male | 2008 | 949 | 36 | 3.8 |
| Male | 2010 | 949 | 26 | 2.7 |
| Male | 2012 | 949 | 10 | 1.1 |
| Male | 2014 | 949 | 9 | 0.9 |
| Female | All waves | 12,593 | 323 | 2.6 |
| Male | All waves | 7,933 | 328 | 4.1 |

*Table A1.3a.* Part-time employment among employed (job-secure/insecure) person-waves, by gender

|  | **Employed person-waves** | **Part-time person-waves** | **% part-time** |
| --- | --- | --- | --- |
| Female | 4,146 | 719 | 17.3 |
| Male | 2,829 | 138 | 4.9 |
| Overall | 6,975 | 857 | 12.3 |

*Table A1.3b.* Distribution of part-time work across individual sequences, among persons ever employed

|  | **N persons ever employed** | **% with any part-time** | **Mean % of employed waves part-time** |
| --- | --- | --- | --- |
| Female | 947 | 35.9 | 18.4 |
| Male | 651 | 14.4 | 4.8 |
| Overall | 1,598 | 27.2 | 12.9 |

*Table A1.4.* Sequence analysis fit statistics for k=2 to k=15 clusters, stratified by gender

| **Men** | | | | | | |
| --- | --- | --- | --- | --- | --- | --- |
| **k** | **PBC** | **HG** | **ASW** | **CH** | **R^2^** | **HC** |
| 2 | 0.438 | 0.593 | 0.309 | 147.1 | 0.134 | 0.209 |
| 3 | 0.632 | 0.784 | 0.385 | 242.1 | 0.339 | 0.118 |
| 4 | 0.586 | 0.762 | 0.310 | 200.9 | 0.389 | 0.128 |
| 5 | 0.625 | 0.856 | 0.338 | 186.2 | 0.441 | 0.091 |
| 6 | 0.559 | 0.835 | 0.309 | 174.4 | 0.481 | 0.104 |
| 7 | 0.497 | 0.795 | 0.264 | 155.0 | 0.497 | 0.134 |
| 8 | 0.486 | 0.790 | 0.228 | 140.6 | 0.511 | 0.138 |
| 9 | 0.461 | 0.787 | 0.198 | 129.4 | 0.524 | 0.146 |
| 10 | 0.468 | 0.813 | 0.211 | 121.7 | 0.538 | 0.133 |
| 11 | 0.465 | 0.826 | 0.221 | 114.5 | 0.550 | 0.127 |
| 12 | 0.476 | 0.857 | 0.243 | 108.0 | 0.559 | 0.111 |
| 13 | 0.452 | 0.848 | 0.219 | 103.5 | 0.570 | 0.116 |
| 14 | 0.449 | 0.859 | 0.219 | 99.1 | 0.579 | 0.109 |
| 15 | 0.447 | 0.868 | 0.223 | 94.5 | 0.586 | 0.102 |
| **Women** | | | | | | |
| **k** | **PBC** | **HG** | **ASW** | **CH** | **R^2^** | **HC** |
| 2 | 0.508 | 0.628 | 0.323 | 360.3 | 0.197 | 0.196 |
| 3 | 0.517 | 0.656 | 0.309 | 306.8 | 0.295 | 0.184 |
| 4 | 0.490 | 0.651 | 0.278 | 272.9 | 0.358 | 0.184 |
| 5 | 0.579 | 0.809 | 0.324 | 276.4 | 0.43 | 0.115 |
| 6 | 0.600 | 0.867 | 0.337 | 256.4 | 0.467 | 0.087 |
| 7 | 0.551 | 0.865 | 0.293 | 240.0 | 0.496 | 0.087 |
| 8 | 0.534 | 0.880 | 0.292 | 228.0 | 0.522 | 0.084 |
| 9 | 0.480 | 0.846 | 0.260 | 208.6 | 0.533 | 0.111 |
| 10 | 0.475 | 0.860 | 0.259 | 194.3 | 0.545 | 0.105 |
| 11 | 0.475 | 0.873 | 0.256 | 184.1 | 0.558 | 0.099 |
| 12 | 0.460 | 0.874 | 0.214 | 174.3 | 0.568 | 0.100 |
| 13 | 0.449 | 0.866 | 0.197 | 164.5 | 0.575 | 0.103 |
| 14 | 0.438 | 0.869 | 0.184 | 156.8 | 0.583 | 0.100 |
| 15 | 0.427 | 0.859 | 0.168 | 146.5 | 0.585 | 0.105 |

*Figure A1.1.* Sequence analysis fit statistics for k=2 to k=15 clusters, stratified by gender

*Table A1.5.* Sensitivity of the six-cluster solution to the dissimilarity specification, by sex

|  | **ARI vs. primary (Men)** | **ARI vs. primary (Women)** | **Archetype recovery (Men)** | **Archetype recovery (Women)** |
| --- | --- | --- | --- | --- |
| Constant cost,  indel = 1 (primary) | 1.000 | 1.000 | 100% | 100% |
| TRATE cost,  indel = 1 | 0.773 | 0.574 | 89% | 82% |
| Constant cost,  indel = 2 | 0.479 | 0.487 | 70% | 72% |
| Constant cost,  indel = 0.5 | 1.000 | 1.000 | 100% | 100% |
| OMspell  (constant cost, indel = 1) | 0.618 | 0.534 | 81% | 77% |
| OMslen  (constant cost, indel = 1) | 0.745 | 0.600 | 88% | 79% |
| LCS (longest common subsequence) | 1.000 | 1.000 | 100% | 100% |

Note. Values are the adjusted Rand index (ARI) and archetype recovery relative to the primary solution. Archetype recovery is the percentage of individuals retaining their primary trajectory archetype under the alternative specification. OM = optimal matching; TRATE = transition-rate substitution costs; OMspell = spell-based OM; OMslen = spell-length–sensitive OM; LCS = longest common subsequence; indel = insertion/deletion cost.

**Appendix 2. Censoring Descriptives and Inverse Probability of Censoring Weight Models**

*Table A2.1.* Censoring status and follow-up among study-eligible participants, overall and by sex

|  | **N** | **Died before 2016** | **Attrited before 2016** | **Retained to 2016** | **Mean follow-up (yrs)** | **Median follow-up (yrs)** |
| --- | --- | --- | --- | --- | --- | --- |
| Overall | 6,672 | 803 (12.0%) | 1502 (22.5%) | 4367 (65.5%) | 14.5 | 12 |
| Female | 3,630 | 348 (9.6%) | 796 (21.9%) | 2486 (68.5%) | 14.5 | 12 |
| Male | 3,042 | 455 (15.0%) | 706 (23.2%) | 1881 (61.8%) | 14.5 | 12 |

Eligible = HRS participants enrolled during the study window (1998-2008) and younger than 65 at baseline. “Retained to 2016” denotes alive and observed in the 2016 wave, from which the analytic sample was drawn; the remainder were censored by death or attrition (attrition includes failure to reach the ≥4-wave requirement). Follow-up is the interval from the baseline wave to the 2016 HCAP.

*Table A2.2.* Mortality and attrition weight models (pooled odds ratios across 20 imputations)

| **Model** | **Term** | **OR (95% CI)** | **p** |
| --- | --- | --- | --- |
| Mortality (died by 2016) | (Intercept) | 0.50 (0.41, 0.60) | <0.001 |
| Mortality (died by 2016) | ragenderFemale | 0.46 (0.42, 0.50) | <0.001 |
| Mortality (died by 2016) | rabplaceEast South Central | 0.95 (0.81, 1.12) | 0.535 |
| Mortality (died by 2016) | rabplaceMid Atlantic | 1.01 (0.87, 1.17) | 0.907 |
| Mortality (died by 2016) | rabplaceMountain | 0.77 (0.60, 0.98) | 0.031 |
| Mortality (died by 2016) | rabplaceNew England | 1.01 (0.81, 1.26) | 0.933 |
| Mortality (died by 2016) | rabplaceOutside US | 0.49 (0.42, 0.59) | <0.001 |
| Mortality (died by 2016) | rabplacePacific | 0.73 (0.60, 0.89) | 0.002 |
| Mortality (died by 2016) | rabplaceSouth Atlantic | 1.03 (0.90, 1.18) | 0.660 |
| Mortality (died by 2016) | rabplaceUS No State | 0.31 (0.07, 1.47) | 0.141 |
| Mortality (died by 2016) | rabplaceWest North Central | 0.91 (0.77, 1.07) | 0.261 |
| Mortality (died by 2016) | rabplaceWest South Central | 0.96 (0.83, 1.12) | 0.630 |
| Mortality (died by 2016) | raeducHS/GED | 0.90 (0.80, 1.00) | 0.060 |
| Mortality (died by 2016) | raeducSome College | 0.93 (0.81, 1.06) | 0.250 |
| Mortality (died by 2016) | raeducCollege+ | 0.79 (0.68, 0.92) | 0.002 |
| Mortality (died by 2016) | mstatSeparated/Divorced | 1.26 (1.12, 1.43) | <0.001 |
| Mortality (died by 2016) | mstatWidowed | 1.38 (1.14, 1.67) | <0.001 |
| Mortality (died by 2016) | mstatNever Married | 1.15 (0.93, 1.43) | 0.204 |
| Mortality (died by 2016) | lbrfWorks PT | 1.10 (0.96, 1.27) | 0.180 |
| Mortality (died by 2016) | lbrfUnemployed | 1.54 (1.25, 1.89) | <0.001 |
| Mortality (died by 2016) | lbrfRetired | 1.66 (1.47, 1.88) | <0.001 |
| Mortality (died by 2016) | lbrfDisabled | 1.45 (1.19, 1.77) | <0.001 |
| Mortality (died by 2016) | lbrfNot in Labor Force | 1.25 (1.08, 1.44) | 0.003 |
| Mortality (died by 2016) | atotw_quint2 | 0.81 (0.72, 0.92) | <0.001 |
| Mortality (died by 2016) | atotw_quint3 | 0.69 (0.61, 0.79) | <0.001 |
| Mortality (died by 2016) | atotw_quint4 | 0.68 (0.59, 0.78) | <0.001 |
| Mortality (died by 2016) | atotw_quint5 | 0.62 (0.53, 0.72) | <0.001 |
| Mortality (died by 2016) | cogtot_z | 0.91 (0.87, 0.96) | <0.001 |
| Mortality (died by 2016) | cesd | 1.01 (0.99, 1.03) | 0.312 |
| Mortality (died by 2016) | mobility1 | 1.36 (1.23, 1.51) | <0.001 |
| Mortality (died by 2016) | mobility2 | 1.52 (1.33, 1.75) | <0.001 |
| Mortality (died by 2016) | mobility3 | 1.83 (1.54, 2.17) | <0.001 |
| Mortality (died by 2016) | mobility4 | 2.01 (1.64, 2.45) | <0.001 |
| Mortality (died by 2016) | mobility5 | 1.96 (1.53, 2.52) | <0.001 |
| Mortality (died by 2016) | shlt2 | 1.23 (1.08, 1.39) | 0.001 |
| Mortality (died by 2016) | shlt3 | 1.64 (1.45, 1.86) | <0.001 |
| Mortality (died by 2016) | shlt4 | 2.38 (2.05, 2.77) | <0.001 |
| Mortality (died by 2016) | shlt5 | 2.35 (1.92, 2.88) | <0.001 |
| Mortality (died by 2016) | proxy1 | 0.94 (0.79, 1.12) | 0.478 |
| Mortality (died by 2016) | baseline_wave2 | 0.67 (0.42, 1.05) | 0.082 |
| Mortality (died by 2016) | baseline_wave3 | 1.13 (0.78, 1.63) | 0.511 |
| Mortality (died by 2016) | baseline_wave4 | 0.42 (0.37, 0.47) | <0.001 |
| Mortality (died by 2016) | baseline_wave5 | 0.67 (0.46, 0.97) | 0.034 |
| Mortality (died by 2016) | baseline_wave6 | 0.39 (0.25, 0.61) | <0.001 |
| Mortality (died by 2016) | baseline_wave7 | 0.18 (0.15, 0.20) | <0.001 |
| Mortality (died by 2016) | baseline_wave8 | 0.21 (0.12, 0.37) | <0.001 |
| Mortality (died by 2016) | baseline_wave9 | 0.20 (0.10, 0.40) | <0.001 |
| Attrition (alive, not in HCAP 2016) | (Intercept) | 0.15 (0.13, 0.19) | <0.001 |
| Attrition (alive, not in HCAP 2016) | ragenderFemale | 1.05 (0.96, 1.15) | 0.253 |
| Attrition (alive, not in HCAP 2016) | rabplaceEast South Central | 1.06 (0.90, 1.26) | 0.490 |
| Attrition (alive, not in HCAP 2016) | rabplaceMid Atlantic | 1.10 (0.95, 1.27) | 0.199 |
| Attrition (alive, not in HCAP 2016) | rabplaceMountain | 1.23 (0.98, 1.54) | 0.071 |
| Attrition (alive, not in HCAP 2016) | rabplaceNew England | 1.27 (1.04, 1.57) | 0.022 |
| Attrition (alive, not in HCAP 2016) | rabplaceOutside US | 1.42 (1.22, 1.65) | <0.001 |
| Attrition (alive, not in HCAP 2016) | rabplacePacific | 1.31 (1.09, 1.56) | 0.003 |
| Attrition (alive, not in HCAP 2016) | rabplaceSouth Atlantic | 1.14 (1.00, 1.31) | 0.058 |
| Attrition (alive, not in HCAP 2016) | rabplaceUS No State | 0.90 (0.30, 2.69) | 0.852 |
| Attrition (alive, not in HCAP 2016) | rabplaceWest North Central | 0.89 (0.75, 1.05) | 0.181 |
| Attrition (alive, not in HCAP 2016) | rabplaceWest South Central | 1.20 (1.02, 1.40) | 0.026 |
| Attrition (alive, not in HCAP 2016) | raeducHS/GED | 1.22 (1.07, 1.37) | 0.002 |
| Attrition (alive, not in HCAP 2016) | raeducSome College | 1.10 (0.95, 1.26) | 0.194 |
| Attrition (alive, not in HCAP 2016) | raeducCollege+ | 1.06 (0.91, 1.23) | 0.463 |
| Attrition (alive, not in HCAP 2016) | mstatSeparated/Divorced | 1.05 (0.92, 1.20) | 0.451 |
| Attrition (alive, not in HCAP 2016) | mstatWidowed | 0.93 (0.75, 1.16) | 0.543 |
| Attrition (alive, not in HCAP 2016) | mstatNever Married | 1.32 (1.07, 1.63) | 0.010 |
| Attrition (alive, not in HCAP 2016) | lbrfWorks PT | 0.98 (0.86, 1.11) | 0.719 |
| Attrition (alive, not in HCAP 2016) | lbrfUnemployed | 0.99 (0.80, 1.24) | 0.956 |
| Attrition (alive, not in HCAP 2016) | lbrfRetired | 0.89 (0.78, 1.02) | 0.091 |
| Attrition (alive, not in HCAP 2016) | lbrfDisabled | 1.05 (0.83, 1.34) | 0.687 |
| Attrition (alive, not in HCAP 2016) | lbrfNot in Labor Force | 1.24 (1.09, 1.43) | 0.002 |
| Attrition (alive, not in HCAP 2016) | atotw_quint2 | 1.11 (0.97, 1.27) | 0.115 |
| Attrition (alive, not in HCAP 2016) | atotw_quint3 | 1.28 (1.11, 1.47) | <0.001 |
| Attrition (alive, not in HCAP 2016) | atotw_quint4 | 1.37 (1.19, 1.57) | <0.001 |
| Attrition (alive, not in HCAP 2016) | atotw_quint5 | 1.37 (1.19, 1.58) | <0.001 |
| Attrition (alive, not in HCAP 2016) | cogtot_z | 1.01 (0.96, 1.06) | 0.798 |
| Attrition (alive, not in HCAP 2016) | cesd | 0.98 (0.96, 1.00) | 0.078 |
| Attrition (alive, not in HCAP 2016) | mobility1 | 0.80 (0.72, 0.89) | <0.001 |
| Attrition (alive, not in HCAP 2016) | mobility2 | 0.85 (0.73, 0.99) | 0.038 |
| Attrition (alive, not in HCAP 2016) | mobility3 | 0.73 (0.59, 0.90) | 0.004 |
| Attrition (alive, not in HCAP 2016) | mobility4 | 0.61 (0.47, 0.80) | <0.001 |
| Attrition (alive, not in HCAP 2016) | mobility5 | 0.65 (0.47, 0.90) | 0.010 |
| Attrition (alive, not in HCAP 2016) | shlt2 | 1.04 (0.94, 1.16) | 0.442 |
| Attrition (alive, not in HCAP 2016) | shlt3 | 0.95 (0.85, 1.06) | 0.378 |
| Attrition (alive, not in HCAP 2016) | shlt4 | 0.84 (0.72, 0.98) | 0.030 |
| Attrition (alive, not in HCAP 2016) | shlt5 | 1.13 (0.90, 1.41) | 0.302 |
| Attrition (alive, not in HCAP 2016) | proxy1 | 1.66 (1.42, 1.93) | <0.001 |
| Attrition (alive, not in HCAP 2016) | baseline_wave2 | 0.88 (0.55, 1.40) | 0.589 |
| Attrition (alive, not in HCAP 2016) | baseline_wave3 | 1.47 (1.02, 2.13) | 0.039 |
| Attrition (alive, not in HCAP 2016) | baseline_wave4 | 1.24 (1.11, 1.38) | <0.001 |
| Attrition (alive, not in HCAP 2016) | baseline_wave5 | 1.54 (1.11, 2.14) | 0.010 |
| Attrition (alive, not in HCAP 2016) | baseline_wave6 | 1.38 (0.95, 1.99) | 0.090 |
| Attrition (alive, not in HCAP 2016) | baseline_wave7 | 1.24 (1.11, 1.37) | <0.001 |
| Attrition (alive, not in HCAP 2016) | baseline_wave8 | 1.11 (0.75, 1.65) | 0.606 |
| Attrition (alive, not in HCAP 2016) | baseline_wave9 | 1.20 (0.76, 1.92) | 0.432 |

Odds ratios are from the separate stabilized IPCW logistic models, pooled across 20 MICE imputations (Rubin’s rules), estimated on the full baseline cohort used for weight construction.

**Appendix 3. Supplementary Sensitivity and Interpretation Analyses**

This appendix contains the results of the sensitivity analyses evaluating the robustness of the associations between job insecurity trajectory clusters and 2016 cognitive outcomes. Unless otherwise noted, all models use the fully adjusted specification, the Predominantly Job Secure cluster as the reference, and the HCAP survey × inverse probability of censoring weights. Effect estimates are reported as odds ratios (OR) for any cognitive impairment outcome and the standardized regression coefficient (β, in SD units) for the cognitive domain outcomes, each with a 95% confidence interval. * denotes p < 0.05. This appendix also includes an interpretive benchmark that expresses the significant domain associations as equivalent years of cognitive aging (Section 4).

**1. Sensitivity to Cluster Specification: Sequence Truncation and Self-Employment**

We re-estimated the associations under three alternative derivations of the trajectory solution. First, we truncated the exposure window to end in 2012 and in 2010 (rather than 2014) while retaining the six-cluster solution to increase the lag between exposure and outcome. Second, we excluded respondents who were self-employed in at least half of their observed waves and re-derived the solution at five clusters (the self-employed cluster being removed). In each case cluster assignment was updated and models were refit. Estimates were stable in direction and magnitude across all specifications.

*Table A3.1.* Model associations under truncated exposure windows and exclusion of predominantly self-employed respondents, among men

| **Outcome** | **Cluster** | **Primary** | **Truncated**  **1998–2012** | **Truncated**  **1998–2010** | **Excluding Self-Employed** |
| --- | --- | --- | --- | --- | --- |
| Any Impairment | Secure Employment to Retirement | 1.45 (0.78, 2.69) | 1.05 (0.56, 1.94) | 1.09 (0.57, 2.09) | 1.46 (0.78, 2.71) |
| Any Impairment | Predominantly Job Insecure | 1.37 (0.68, 2.77) | 1.20 (0.63, 2.29) | 0.99 (0.52, 1.89) | 1.37 (0.68, 2.76) |
| Any Impairment | Predominantly Self-Employed | 0.89 (0.43, 1.81) | 0.76 (0.38, 1.54) | 0.68 (0.35, 1.34) |  |
| Any Impairment | Early Retirement | 1.48 (0.74, 2.96) | 1.06 (0.55, 2.05) | 1.01 (0.53, 1.92) | 1.60 (0.79, 3.24) |
| Any Impairment | Inconsistent Employment | 2.02 (1.06, 3.83) * | 1.85 (1.05, 3.27) * | 2.00 (1.14, 3.51) * | 2.00 (1.04, 3.85) * |
| Memory | Secure Employment to Retirement | -0.12 (-0.30, 0.05) | -0.05 (-0.23, 0.14) | -0.03 (-0.23, 0.16) | -0.11 (-0.29, 0.06) |
| Memory | Predominantly Job Insecure | -0.04 (-0.25, 0.16) | 0.02 (-0.17, 0.22) | 0.06 (-0.13, 0.24) | -0.03 (-0.24, 0.17) |
| Memory | Predominantly Self-Employed | -0.08 (-0.32, 0.16) | -0.02 (-0.25, 0.22) | 0.01 (-0.21, 0.24) |  |
| Memory | Early Retirement | -0.09 (-0.30, 0.12) | -0.06 (-0.28, 0.16) | -0.04 (-0.25, 0.18) | -0.06 (-0.28, 0.15) |
| Memory | Inconsistent Employment | -0.15 (-0.37, 0.07) | -0.11 (-0.30, 0.09) | -0.12 (-0.32, 0.09) | -0.13 (-0.34, 0.09) |
| Executive Function | Secure Employment to Retirement | 0.00 (-0.16, 0.16) | 0.05 (-0.13, 0.22) | 0.03 (-0.15, 0.22) | 0.01 (-0.16, 0.17) |
| Executive Function | Predominantly Job Insecure | 0.04 (-0.13, 0.21) | 0.03 (-0.13, 0.20) | -0.00 (-0.17, 0.16) | 0.04 (-0.14, 0.22) |
| Executive Function | Predominantly Self-Employed | -0.01 (-0.19, 0.17) | 0.01 (-0.16, 0.18) | 0.02 (-0.14, 0.19) |  |
| Executive Function | Early Retirement | -0.15 (-0.36, 0.05) | -0.04 (-0.24, 0.17) | -0.04 (-0.24, 0.17) | -0.16 (-0.36, 0.05) |
| Executive Function | Inconsistent Employment | -0.20 (-0.39, -0.00) * | -0.27 (-0.46, -0.09) * | -0.30 (-0.49, -0.11) * | -0.18 (-0.37, 0.02) |
| Language & Fluency | Secure Employment to Retirement | 0.04 (-0.22, 0.30) | 0.06 (-0.20, 0.32) | 0.01 (-0.25, 0.27) | 0.06 (-0.20, 0.31) |
| Language & Fluency | Predominantly Job Insecure | -0.30 (-0.56, -0.03) * | -0.30 (-0.54, -0.05) * | -0.15 (-0.41, 0.11) | -0.29 (-0.55, -0.03) * |
| Language & Fluency | Predominantly Self-Employed | -0.09 (-0.36, 0.18) | -0.06 (-0.32, 0.21) | -0.03 (-0.28, 0.23) |  |
| Language & Fluency | Early Retirement | -0.14 (-0.40, 0.12) | -0.14 (-0.40, 0.13) | -0.13 (-0.39, 0.13) | -0.11 (-0.38, 0.16) |
| Language & Fluency | Inconsistent Employment | -0.29 (-0.56, -0.03) * | -0.33 (-0.57, -0.09) * | -0.31 (-0.55, -0.07) * | -0.26 (-0.53, 0.01) |
| Orientation | Secure Employment to Retirement | -0.05 (-0.19, 0.09) | 0.08 (-0.07, 0.23) | 0.07 (-0.09, 0.24) | -0.04 (-0.18, 0.10) |
| Orientation | Predominantly Job Insecure | 0.04 (-0.10, 0.18) | 0.10 (-0.07, 0.26) | 0.11 (-0.05, 0.27) | 0.05 (-0.09, 0.19) |
| Orientation | Predominantly Self-Employed | 0.01 (-0.11, 0.14) | 0.09 (-0.05, 0.24) | 0.10 (-0.04, 0.24) |  |
| Orientation | Early Retirement | -0.05 (-0.22, 0.12) | 0.03 (-0.18, 0.25) | 0.02 (-0.19, 0.23) | -0.05 (-0.23, 0.13) |
| Orientation | Inconsistent Employment | -0.27 (-0.44, -0.10) * | -0.20 (-0.39, -0.02) * | -0.22 (-0.42, -0.03) * | -0.27 (-0.44, -0.10) * |
| Global Cognition | Secure Employment to Retirement | -0.02 (-0.17, 0.14) | 0.02 (-0.13, 0.17) | -0.00 (-0.16, 0.16) | -0.00 (-0.15, 0.15) |
| Global Cognition | Predominantly Job Insecure | -0.06 (-0.23, 0.11) | -0.03 (-0.19, 0.13) | -0.04 (-0.19, 0.12) | -0.05 (-0.22, 0.13) |
| Global Cognition | Predominantly Self-Employed | -0.08 (-0.26, 0.09) | -0.06 (-0.22, 0.11) | -0.07 (-0.23, 0.09) |  |
| Global Cognition | Early Retirement | -0.25 (-0.44, -0.06) * | -0.15 (-0.33, 0.03) | -0.15 (-0.33, 0.03) | -0.23 (-0.42, -0.03) * |
| Global Cognition | Inconsistent Employment | -0.29 (-0.49, -0.09) * | -0.35 (-0.54, -0.16) * | -0.39 (-0.59, -0.20) * | -0.27 (-0.46, -0.07) * |

*Table A3.2.* Model associations under truncated exposure windows and exclusion of predominantly self-employed respondents, among women

| **Outcome** | **Cluster** | **Primary** | **Truncated 1998–2012** | **Truncated 1998–2010** | **Excluding Self-Employed** |
| --- | --- | --- | --- | --- | --- |
| Any Impairment | Secure Employment to Retirement | 1.36 (0.83, 2.24) | 1.42 (0.86, 2.33) | 1.32 (0.82, 2.10) | 1.45 (0.88, 2.39) |
| Any Impairment | Predominantly Job Insecure | 0.46 (0.23, 0.92) * | 0.45 (0.23, 0.90) * | 0.48 (0.25, 0.92) * | 0.47 (0.23, 0.95) * |
| Any Impairment | Predominantly Self-Employed | 1.87 (0.98, 3.59) | 1.84 (0.96, 3.53) | 2.05 (1.13, 3.73) * |  |
| Any Impairment | Early Retirement | 1.97 (1.19, 3.27) * | 1.93 (1.18, 3.17) * | 1.88 (1.16, 3.03) * | 2.00 (1.20, 3.34) * |
| Any Impairment | Not in Labor Force | 1.80 (1.03, 3.16) * | 1.86 (1.07, 3.26) * | 1.73 (1.02, 2.91) * | 1.89 (1.07, 3.32) * |
| Memory | Secure Employment to Retirement | -0.19 (-0.33, -0.05) * | -0.20 (-0.34, -0.06) * | -0.20 (-0.34, -0.06) * | -0.18 (-0.32, -0.04) * |
| Memory | Predominantly Job Insecure | -0.02 (-0.19, 0.15) | -0.07 (-0.23, 0.09) | -0.01 (-0.17, 0.16) | -0.03 (-0.20, 0.14) |
| Memory | Predominantly Self-Employed | -0.24 (-0.42, -0.07) * | -0.25 (-0.43, -0.07) * | -0.23 (-0.40, -0.06) * |  |
| Memory | Early Retirement | -0.30 (-0.46, -0.13) * | -0.30 (-0.46, -0.15) * | -0.27 (-0.43, -0.11) * | -0.29 (-0.46, -0.12) * |
| Memory | Not in Labor Force | -0.32 (-0.50, -0.14) * | -0.33 (-0.50, -0.16) * | -0.27 (-0.44, -0.10) * | -0.32 (-0.50, -0.14) * |
| Executive Function | Secure Employment to Retirement | -0.13 (-0.25, -0.01) * | -0.12 (-0.24, 0.01) | -0.10 (-0.22, 0.02) | -0.12 (-0.25, -0.00) * |
| Executive Function | Predominantly Job Insecure | -0.01 (-0.16, 0.14) | -0.05 (-0.19, 0.10) | 0.01 (-0.13, 0.15) | -0.02 (-0.17, 0.13) |
| Executive Function | Predominantly Self-Employed | -0.15 (-0.30, -0.01) * | -0.14 (-0.30, 0.01) | -0.14 (-0.30, 0.02) |  |
| Executive Function | Early Retirement | -0.21 (-0.35, -0.07) * | -0.19 (-0.32, -0.05) * | -0.16 (-0.30, -0.02) * | -0.21 (-0.35, -0.06) * |
| Executive Function | Not in Labor Force | -0.25 (-0.39, -0.11) * | -0.27 (-0.41, -0.12) * | -0.24 (-0.38, -0.10) * | -0.24 (-0.38, -0.10) * |
| Language & Fluency | Secure Employment to Retirement | -0.04 (-0.21, 0.13) | -0.10 (-0.27, 0.08) | -0.15 (-0.32, 0.02) | -0.06 (-0.23, 0.11) |
| Language & Fluency | Predominantly Job Insecure | 0.12 (-0.09, 0.33) | 0.02 (-0.17, 0.21) | 0.01 (-0.18, 0.20) | 0.10 (-0.10, 0.31) |
| Language & Fluency | Predominantly Self-Employed | -0.29 (-0.50, -0.08) * | -0.30 (-0.51, -0.08) * | -0.30 (-0.51, -0.09) * |  |
| Language & Fluency | Early Retirement | -0.14 (-0.33, 0.04) | -0.15 (-0.34, 0.03) | -0.16 (-0.35, 0.03) | -0.15 (-0.33, 0.04) |
| Language & Fluency | Not in Labor Force | -0.13 (-0.34, 0.07) | -0.15 (-0.36, 0.05) | -0.14 (-0.35, 0.07) | -0.14 (-0.35, 0.06) |
| Orientation | Secure Employment to Retirement | -0.00 (-0.10, 0.09) | -0.04 (-0.14, 0.06) | -0.00 (-0.10, 0.10) | 0.00 (-0.10, 0.10) |
| Orientation | Predominantly Job Insecure | 0.08 (-0.05, 0.20) | 0.08 (-0.03, 0.18) | 0.06 (-0.06, 0.18) | 0.08 (-0.05, 0.20) |
| Orientation | Predominantly Self-Employed | -0.06 (-0.21, 0.09) | -0.07 (-0.23, 0.09) | -0.11 (-0.26, 0.04) |  |
| Orientation | Early Retirement | -0.17 (-0.33, -0.00) * | -0.16 (-0.31, -0.01) * | -0.22 (-0.40, -0.05) * | -0.16 (-0.32, 0.00) |
| Orientation | Not in Labor Force | -0.19 (-0.34, -0.05) * | -0.22 (-0.37, -0.07) * | -0.23 (-0.37, -0.08) * | -0.19 (-0.33, -0.04) * |
| Global Cognition | Secure Employment to Retirement | -0.10 (-0.21, 0.01) | -0.13 (-0.25, -0.02) * | -0.13 (-0.25, -0.01) * | -0.10 (-0.21, 0.01) |
| Global Cognition | Predominantly Job Insecure | 0.08 (-0.07, 0.22) | 0.06 (-0.07, 0.20) | 0.10 (-0.03, 0.24) | 0.07 (-0.08, 0.21) |
| Global Cognition | Predominantly Self-Employed | -0.19 (-0.36, -0.02) * | -0.20 (-0.37, -0.02) * | -0.20 (-0.36, -0.03) * |  |
| Global Cognition | Early Retirement | -0.18 (-0.32, -0.03) * | -0.16 (-0.30, -0.02) * | -0.14 (-0.29, 0.01) | -0.17 (-0.32, -0.03) * |
| Global Cognition | Not in Labor Force | -0.23 (-0.39, -0.07) * | -0.25 (-0.41, -0.10) * | -0.21 (-0.36, -0.06) * | -0.22 (-0.38, -0.07) * |

**2. Sensitivity to Baseline Inclusion (Prevalent Impairment or Disability)**

Because the original sample did not exclude respondents with evidence of prevalent impairment or disability at study entry, we refit the primary models using three different sets of baseline restrictions while holding cluster assignment fixed. The three restrictions were: Model A = excludes probable baseline dementia and probable baseline disability; Model B = excludes any probable baseline cognitive impairment; and Model C = excludes probable baseline disability. Cognitive impairment was defined by the Langa–Weir classification of the HRS core 27-point cognitive scale where scores ≤ 11 represented any probable cognitive impairment and ≤ 6 represented probable dementia. Probable disability was defined as any reported activity-of-daily-living (ADL) limitation.

*Table A3.3.* Retained sample size by exclusion model

| **Exclusion model** | **Men**  **N retained** | **Women**  **N retained** |
| --- | --- | --- |
| Primary | 948 | 1470 |
| A: Excl. dementia or disability | 884 | 1322 |
| B: Excl. any cognitive impairment | 878 | 1357 |
| C: Excl. disability | 889 | 1329 |

*Table A3.4.* Model associations across baseline-inclusion samples, among men

| **Outcome** | **Cluster** | **Primary** | **Model A** | **Model B** | **Model C** |
| --- | --- | --- | --- | --- | --- |
| Any Impairment | Secure Employment to Retirement | 1.45 (0.78, 2.69) | 1.47 (0.79, 2.75) | 1.40 (0.75, 2.62) | 1.49 (0.80, 2.78) |
| Any Impairment | Predominantly Job Insecure | 1.37 (0.68, 2.77) | 1.38 (0.68, 2.81) | 1.43 (0.70, 2.92) | 1.33 (0.65, 2.71) |
| Any Impairment | Predominantly Self-Employed | 0.89 (0.43, 1.81) | 0.86 (0.41, 1.79) | 0.80 (0.38, 1.68) | 0.86 (0.42, 1.79) |
| Any Impairment | Early Retirement | 1.48 (0.74, 2.96) | 1.59 (0.79, 3.21) | 1.59 (0.80, 3.18) | 1.59 (0.79, 3.20) |
| Any Impairment | Inconsistent Employment | 2.02 (1.06, 3.83)* | 1.85 (0.94, 3.63) | 1.80 (0.92, 3.51) | 1.86 (0.95, 3.64) |
| Memory | Secure Employment to Retirement | -0.12 (-0.30, 0.05) | -0.12 (-0.30, 0.06) | -0.12 (-0.30, 0.07) | -0.12 (-0.30, 0.06) |
| Memory | Predominantly Job Insecure | -0.04 (-0.25, 0.16) | -0.03 (-0.23, 0.17) | -0.05 (-0.26, 0.16) | -0.02 (-0.22, 0.18) |
| Memory | Predominantly Self-Employed | -0.08 (-0.32, 0.16) | -0.07 (-0.31, 0.17) | -0.07 (-0.31, 0.17) | -0.07 (-0.31, 0.17) |
| Memory | Early Retirement | -0.09 (-0.30, 0.12) | -0.08 (-0.30, 0.14) | -0.12 (-0.34, 0.09) | -0.08 (-0.30, 0.14) |
| Memory | Inconsistent Employment | -0.15 (-0.37, 0.07) | -0.16 (-0.39, 0.08) | -0.12 (-0.35, 0.10) | -0.16 (-0.39, 0.08) |
| Executive Function | Secure Employment to Retirement | 0.00 (-0.16, 0.16) | -0.01 (-0.17, 0.16) | 0.03 (-0.13, 0.19) | -0.01 (-0.17, 0.16) |
| Executive Function | Predominantly Job Insecure | 0.04 (-0.13, 0.21) | 0.01 (-0.16, 0.19) | 0.02 (-0.16, 0.19) | 0.02 (-0.16, 0.20) |
| Executive Function | Predominantly Self-Employed | -0.01 (-0.19, 0.17) | -0.01 (-0.19, 0.17) | -0.00 (-0.18, 0.18) | -0.01 (-0.19, 0.16) |
| Executive Function | Early Retirement | -0.15 (-0.36, 0.05) | -0.19 (-0.40, 0.02) | -0.16 (-0.36, 0.04) | -0.19 (-0.41, 0.02) |
| Executive Function | Inconsistent Employment | -0.20 (-0.39, -0.00)* | -0.21 (-0.43, -0.00)* | -0.19 (-0.39, 0.01) | -0.22 (-0.43, -0.01)* |
| Language & Fluency | Secure Employment to Retirement | 0.04 (-0.22, 0.30) | 0.04 (-0.23, 0.30) | 0.05 (-0.21, 0.31) | 0.03 (-0.23, 0.29) |
| Language & Fluency | Predominantly Job Insecure | -0.30 (-0.56, -0.03)* | -0.32 (-0.59, -0.05)* | -0.34 (-0.60, -0.07)* | -0.33 (-0.59, -0.06)* |
| Language & Fluency | Predominantly Self-Employed | -0.09 (-0.36, 0.18) | -0.10 (-0.37, 0.17) | -0.08 (-0.35, 0.18) | -0.10 (-0.37, 0.17) |
| Language & Fluency | Early Retirement | -0.14 (-0.40, 0.12) | -0.19 (-0.46, 0.09) | -0.12 (-0.39, 0.15) | -0.19 (-0.47, 0.08) |
| Language & Fluency | Inconsistent Employment | -0.29 (-0.56, -0.03)* | -0.30 (-0.58, -0.02)* | -0.28 (-0.55, -0.00)* | -0.30 (-0.58, -0.01)* |
| Orientation | Secure Employment to Retirement | -0.05 (-0.19, 0.09) | -0.05 (-0.19, 0.09) | -0.01 (-0.14, 0.12) | -0.06 (-0.20, 0.08) |
| Orientation | Predominantly Job Insecure | 0.04 (-0.10, 0.18) | 0.02 (-0.12, 0.16) | 0.01 (-0.12, 0.15) | 0.03 (-0.11, 0.18) |
| Orientation | Predominantly Self-Employed | 0.01 (-0.11, 0.14) | 0.01 (-0.11, 0.14) | 0.03 (-0.09, 0.15) | 0.01 (-0.11, 0.14) |
| Orientation | Early Retirement | -0.05 (-0.22, 0.12) | -0.10 (-0.28, 0.08) | -0.07 (-0.24, 0.10) | -0.10 (-0.28, 0.08) |
| Orientation | Inconsistent Employment | -0.27 (-0.44, -0.10)* | -0.31 (-0.49, -0.12)* | -0.29 (-0.46, -0.12)* | -0.30 (-0.48, -0.12)* |
| Global Cognition | Secure Employment to Retirement | -0.02 (-0.17, 0.14) | -0.02 (-0.17, 0.14) | 0.00 (-0.15, 0.15) | -0.02 (-0.17, 0.14) |
| Global Cognition | Predominantly Job Insecure | -0.06 (-0.23, 0.11) | -0.06 (-0.23, 0.11) | -0.10 (-0.27, 0.08) | -0.05 (-0.23, 0.12) |
| Global Cognition | Predominantly Self-Employed | -0.08 (-0.26, 0.09) | -0.07 (-0.25, 0.10) | -0.08 (-0.26, 0.10) | -0.08 (-0.26, 0.10) |
| Global Cognition | Early Retirement | -0.25 (-0.44, -0.06)* | -0.25 (-0.46, -0.05)* | -0.26 (-0.45, -0.07)* | -0.26 (-0.46, -0.05)* |
| Global Cognition | Inconsistent Employment | -0.29 (-0.49, -0.09)* | -0.28 (-0.50, -0.07)* | -0.28 (-0.48, -0.08)* | -0.29 (-0.50, -0.07)* |

*Table A3.5.* Model associations across baseline-inclusion samples, among women

| **Outcome** | **Cluster** | **Primary** | **Model A** | **Model B** | **Model C** |
| --- | --- | --- | --- | --- | --- |
| Any Impairment | Secure Employment to Retirement | 1.36 (0.83, 2.24) | 1.27 (0.76, 2.12) | 1.44 (0.86, 2.39) | 1.26 (0.75, 2.10) |
| Any Impairment | Predominantly Job Insecure | 0.46 (0.23, 0.92)* | 0.48 (0.23, 1.00) | 0.46 (0.22, 0.97)* | 0.48 (0.23, 0.99)* |
| Any Impairment | Predominantly Self-Employed | 1.87 (0.98, 3.59) | 1.85 (0.96, 3.58) | 1.96 (1.00, 3.84)* | 1.86 (0.96, 3.62) |
| Any Impairment | Early Retirement | 1.97 (1.19, 3.27)* | 1.67 (0.98, 2.83) | 2.05 (1.22, 3.45)* | 1.68 (0.99, 2.86) |
| Any Impairment | Not in Labor Force | 1.80 (1.03, 3.16)* | 1.62 (0.90, 2.93) | 1.46 (0.80, 2.66) | 1.69 (0.94, 3.04) |
| Memory | Secure Employment to Retirement | -0.19 (-0.33, -0.05)* | -0.19 (-0.33, -0.05)* | -0.18 (-0.32, -0.04)* | -0.19 (-0.33, -0.04)* |
| Memory | Predominantly Job Insecure | -0.02 (-0.19, 0.15) | -0.04 (-0.21, 0.13) | -0.04 (-0.21, 0.14) | -0.04 (-0.21, 0.13) |
| Memory | Predominantly Self-Employed | -0.24 (-0.42, -0.07)* | -0.24 (-0.42, -0.06)* | -0.25 (-0.43, -0.07)* | -0.24 (-0.42, -0.06)* |
| Memory | Early Retirement | -0.30 (-0.46, -0.13)* | -0.23 (-0.41, -0.06)* | -0.29 (-0.46, -0.12)* | -0.24 (-0.42, -0.07)* |
| Memory | Not in Labor Force | -0.32 (-0.50, -0.14)* | -0.26 (-0.44, -0.09)* | -0.30 (-0.49, -0.11)* | -0.27 (-0.44, -0.09)* |
| Executive Function | Secure Employment to Retirement | -0.13 (-0.25, -0.01)* | -0.13 (-0.25, -0.00)* | -0.13 (-0.26, -0.00)* | -0.12 (-0.25, 0.00) |
| Executive Function | Predominantly Job Insecure | -0.01 (-0.16, 0.14) | -0.03 (-0.18, 0.12) | -0.04 (-0.19, 0.12) | -0.03 (-0.18, 0.12) |
| Executive Function | Predominantly Self-Employed | -0.15 (-0.30, -0.01)* | -0.16 (-0.31, -0.01)* | -0.17 (-0.32, -0.01)* | -0.16 (-0.31, -0.01)* |
| Executive Function | Early Retirement | -0.21 (-0.35, -0.07)* | -0.18 (-0.32, -0.04)* | -0.23 (-0.37, -0.08)* | -0.18 (-0.33, -0.04)* |
| Executive Function | Not in Labor Force | -0.25 (-0.39, -0.11)* | -0.24 (-0.38, -0.09)* | -0.19 (-0.34, -0.05)* | -0.23 (-0.38, -0.09)* |
| Language & Fluency | Secure Employment to Retirement | -0.04 (-0.21, 0.13) | -0.03 (-0.20, 0.15) | -0.03 (-0.20, 0.14) | -0.03 (-0.20, 0.15) |
| Language & Fluency | Predominantly Job Insecure | 0.12 (-0.09, 0.33) | 0.11 (-0.10, 0.33) | 0.14 (-0.08, 0.35) | 0.11 (-0.11, 0.32) |
| Language & Fluency | Predominantly Self-Employed | -0.29 (-0.50, -0.08)* | -0.29 (-0.50, -0.08)* | -0.28 (-0.49, -0.07)* | -0.29 (-0.50, -0.08)* |
| Language & Fluency | Early Retirement | -0.14 (-0.33, 0.04) | -0.12 (-0.31, 0.07) | -0.16 (-0.35, 0.03) | -0.13 (-0.32, 0.06) |
| Language & Fluency | Not in Labor Force | -0.13 (-0.34, 0.07) | -0.08 (-0.29, 0.12) | -0.13 (-0.35, 0.08) | -0.08 (-0.28, 0.12) |
| Orientation | Secure Employment to Retirement | -0.00 (-0.10, 0.09) | -0.01 (-0.11, 0.09) | -0.02 (-0.12, 0.07) | -0.01 (-0.11, 0.09) |
| Orientation | Predominantly Job Insecure | 0.08 (-0.05, 0.20) | 0.08 (-0.03, 0.18) | 0.05 (-0.07, 0.17) | 0.07 (-0.03, 0.18) |
| Orientation | Predominantly Self-Employed | -0.06 (-0.21, 0.09) | -0.06 (-0.21, 0.09) | -0.07 (-0.22, 0.08) | -0.07 (-0.22, 0.08) |
| Orientation | Early Retirement | -0.17 (-0.33, -0.00)* | -0.13 (-0.30, 0.04) | -0.18 (-0.35, -0.01)* | -0.15 (-0.33, 0.02) |
| Orientation | Not in Labor Force | -0.19 (-0.34, -0.05)* | -0.17 (-0.32, -0.02)* | -0.16 (-0.31, -0.01)* | -0.19 (-0.34, -0.03)* |
| Global Cognition | Secure Employment to Retirement | -0.10 (-0.21, 0.01) | -0.10 (-0.21, 0.02) | -0.10 (-0.22, 0.02) | -0.09 (-0.21, 0.03) |
| Global Cognition | Predominantly Job Insecure | 0.08 (-0.07, 0.22) | 0.03 (-0.10, 0.17) | 0.05 (-0.10, 0.20) | 0.04 (-0.10, 0.18) |
| Global Cognition | Predominantly Self-Employed | -0.19 (-0.36, -0.02)* | -0.19 (-0.36, -0.03)* | -0.20 (-0.37, -0.03)* | -0.20 (-0.36, -0.03)* |
| Global Cognition | Early Retirement | -0.18 (-0.32, -0.03)* | -0.15 (-0.29, 0.00) | -0.20 (-0.35, -0.05)* | -0.15 (-0.30, -0.01)* |
| Global Cognition | Not in Labor Force | -0.23 (-0.39, -0.07)* | -0.16 (-0.31, -0.01)* | -0.16 (-0.32, 0.00) | -0.17 (-0.32, -0.02)* |

**3. E-Values for Residual Confounding**

E-values (VanderWeele & Ding, 2017) quantify the minimum strength of association on the risk-ratio scale that an unmeasured confounder would need to have with both the job insecurity trajectory cluster and the cognitive outcome (in addition to all measured covariates) to fully explain away the observed association or to shift its 95% confidence interval to include the null (E-value [CI]). Values are shown for the statistically significant associations from the primary models.

*Table A3.6.* E-values for the significant fully adjusted associations

| **Gender** | **Outcome** | **Cluster** | **Scale** | **Estimate (95% CI)** | **E-value** | **E-value (CI)** |
| --- | --- | --- | --- | --- | --- | --- |
| Men | Any Impairment | Inconsistent Employment | OR | 2.02 (1.06, 3.83) | 2.19 | 1.21 |
| Men | Executive Function | Inconsistent Employment | β (SD) | -0.20 (-0.39, -0.00) | 1.89 | 1.05 |
| Men | Language & Fluency | Predominantly Job Insecure | β (SD) | -0.30 (-0.56, -0.03) | 2.06 | 1.22 |
| Men | Language & Fluency | Inconsistent Employment | β (SD) | -0.29 (-0.56, -0.03) | 2.05 | 1.19 |
| Men | Orientation | Inconsistent Employment | β (SD) | -0.27 (-0.44, -0.10) | 2.19 | 1.53 |
| Men | Global Cognition | Early Retirement | β (SD) | -0.25 (-0.44, -0.06) | 2.16 | 1.38 |
| Men | Global Cognition | Inconsistent Employment | β (SD) | -0.29 (-0.49, -0.09) | 2.33 | 1.52 |
| Women | Any Impairment | Predominantly Job Insecure | OR | 0.46 (0.23, 0.92) | 2.33 | 1.25 |
| Women | Any Impairment | Early Retirement | OR | 1.97 (1.19, 3.27) | 2.16 | 1.40 |
| Women | Any Impairment | Not in Labor Force | OR | 1.80 (1.03, 3.16) | 2.02 | 1.14 |
| Women | Memory | Secure Employment to Retirement | β (SD) | -0.19 (-0.33, -0.05) | 1.76 | 1.29 |
| Women | Memory | Predominantly Self-Employed | β (SD) | -0.24 (-0.42, -0.07) | 1.94 | 1.36 |
| Women | Memory | Early Retirement | β (SD) | -0.30 (-0.46, -0.13) | 2.12 | 1.59 |
| Women | Memory | Not in Labor Force | β (SD) | -0.32 (-0.50, -0.14) | 2.21 | 1.62 |
| Women | Executive Function | Secure Employment to Retirement | β (SD) | -0.13 (-0.25, -0.01) | 1.64 | 1.12 |
| Women | Executive Function | Predominantly Self-Employed | β (SD) | -0.15 (-0.30, -0.01) | 1.73 | 1.09 |
| Women | Executive Function | Early Retirement | β (SD) | -0.21 (-0.35, -0.07) | 1.93 | 1.40 |
| Women | Executive Function | Not in Labor Force | β (SD) | -0.25 (-0.39, -0.11) | 2.07 | 1.55 |
| Women | Language & Fluency | Predominantly Self-Employed | β (SD) | -0.29 (-0.50, -0.08) | 2.03 | 1.40 |
| Women | Orientation | Early Retirement | β (SD) | -0.17 (-0.33, -0.00) | 1.70 | 1.07 |
| Women | Orientation | Not in Labor Force | β (SD) | -0.19 (-0.34, -0.05) | 1.79 | 1.30 |
| Women | Global Cognition | Predominantly Self-Employed | β (SD) | -0.19 (-0.36, -0.02) | 1.84 | 1.19 |
| Women | Global Cognition | Early Retirement | β (SD) | -0.18 (-0.32, -0.03) | 1.79 | 1.24 |
| Women | Global Cognition | Not in Labor Force | β (SD) | -0.23 (-0.39, -0.07) | 1.98 | 1.41 |

**4. Effect-Size Benchmark: Equivalent Years of Cognitive Aging**

To aid interpretation of the domain-score associations, each statistically significant cluster coefficient was expressed as the equivalent number of years of cognitive aging. We calculated this by dividing the coefficient by the within-model annual age coefficient for the same domain and sex (both in SD units). Larger values indicate a greater equivalent age difference relative to the Predominantly Job Secure reference cluster. The language & fluency and orientation domains have shallow age slopes, so their year-equivalents are correspondingly large, unstable, and should be interpreted with caution. Orientation is age-insensitive as it is near its ceiling in cognitively unimpaired adults, with little age-related variation until later-life. Language & fluency reflects crystallized abilities that are relatively preserved with age. Because the age slope is the denominator of the benchmark, shallow gradients yield large and unstable year-equivalents. The benchmark is most reliable for domains with steeper, more stable age gradients like memory, executive function, and global cognition. Finally, the benchmark is a between-person age-equivalence based on cross-sectional age differences at the 2016 assessment. It does not represent within-person cognitive decline over time.

*Table A3.7.* Cognitive-domain associations expressed as equivalent years of cognitive aging

| **Gender** | **Cluster** | **Domain** | **β (95% CI)** | **Age Slope (z/yr)** | **Years of Cognitive Aging** |
| --- | --- | --- | --- | --- | --- |
| Men | Early Retirement | Global Cognition | -0.25 (-0.44, -0.06) | -0.041 | 6.0 (1.4, 10.7) |
| Men | Inconsistent Employment | Executive Function | -0.20 (-0.39, -0.00) | -0.057 | 3.4 (0.0, 6.8) |
| Men | Inconsistent Employment | Global Cognition | -0.29 (-0.49, -0.09) | -0.041 | 7.0 (2.2, 11.7) |
| Men | Inconsistent Employment | Language & Fluency | -0.29 (-0.56, -0.03) | -0.022 | 13.2 (1.2, 25.3) |
| Men | Inconsistent Employment | Orientation | -0.27 (-0.44, -0.10) | -0.022 | 12.0 (4.4, 19.5) |
| Men | Predominantly Job Insecure | Language & Fluency | -0.30 (-0.56, -0.03) | -0.022 | 13.5 (1.5, 25.4) |
| Women | Early Retirement | Executive Function | -0.21 (-0.35, -0.07) | -0.067 | 3.1 (1.0, 5.2) |
| Women | Early Retirement | Global Cognition | -0.18 (-0.32, -0.03) | -0.059 | 3.0 (0.5, 5.4) |
| Women | Early Retirement | Memory | -0.30 (-0.46, -0.13) | -0.050 | 5.9 (2.6, 9.1) |
| Women | Early Retirement | Orientation | -0.17 (-0.33, -0.00) | -0.038 | 4.3 (0.1, 8.6) |
| Women | Not in Labor Force | Executive Function | -0.25 (-0.39, -0.11) | -0.067 | 3.7 (1.6, 5.8) |
| Women | Not in Labor Force | Global Cognition | -0.23 (-0.39, -0.07) | -0.059 | 3.9 (1.2, 6.5) |
| Women | Not in Labor Force | Memory | -0.32 (-0.50, -0.14) | -0.050 | 6.4 (2.8, 10.0) |
| Women | Not in Labor Force | Orientation | -0.19 (-0.34, -0.05) | -0.038 | 5.1 (1.3, 8.9) |
| Women | Predominantly Self-Employed | Executive Function | -0.15 (-0.30, -0.01) | -0.067 | 2.3 (0.1, 4.5) |
| Women | Predominantly Self-Employed | Global Cognition | -0.19 (-0.36, -0.02) | -0.059 | 3.2 (0.4, 6.0) |
| Women | Predominantly Self-Employed | Language & Fluency | -0.29 (-0.50, -0.08) | -0.030 | 9.6 (2.7, 16.4) |
| Women | Predominantly Self-Employed | Memory | -0.24 (-0.42, -0.07) | -0.050 | 4.8 (1.3, 8.4) |
| Women | Secure Employment to Retirement | Executive Function | -0.13 (-0.25, -0.01) | -0.067 | 2.0 (0.1, 3.8) |
| Women | Secure Employment to Retirement | Memory | -0.19 (-0.33, -0.05) | -0.050 | 3.7 (0.9, 6.5) |

Years of cognitive aging = the cluster's standardized regression coefficient (β) divided by the model's annual age coefficient for the same domain and sex (both in SD units); the reported range divides the 95% CI of β by the age slope. All estimates are from the fully adjusted model, with the Predominantly Job Secure cluster as the reference.
